# Identification of Novel and Conserved MicroRNAs Related to Drought Stress in Potato by Deep Sequencing

**DOI:** 10.1371/journal.pone.0095489

**Published:** 2014-04-18

**Authors:** Ning Zhang, Jiangwei Yang, Zemin Wang, Yikai Wen, Jie Wang, Wenhui He, Bailin Liu, Huaijun Si, Di Wang

**Affiliations:** 1 Gansu Key Laboratory of Crop Genetic and Germplasm Enhancement, Gansu Provincial Key Laboratory of Aridland Crop Science, Gansu Agricultural University, Lanzhou, People's Republic of China; 2 College of Life Science and Technology, Gansu Agricultural University, Lanzhou, People's Republic of China; Universidade Federal do Rio Grande do Sul, Brazil

## Abstract

MicroRNAs (miRNAs) are a group of small, non-coding RNAs that play important roles in plant growth, development and stress response. There have been an increasing number of investigations aimed at discovering miRNAs and analyzing their functions in model plants (such as *Arabidopsis thaliana* and rice). In this research, we constructed small RNA libraries from both polyethylene glycol (PEG 6,000) treated and control potato samples, and a large number of known and novel miRNAs were identified. Differential expression analysis showed that 100 of the known miRNAs were down-regulated and 99 were up-regulated as a result of PEG stress, while 119 of the novel miRNAs were up-regulated and 151 were down-regulated. Based on target prediction, annotation and expression analysis of the miRNAs and their putative target genes, 4 miRNAs were identified as regulating drought-related genes (miR811, miR814, miR835, miR4398). Their target genes were MYB transcription factor (CV431094), hydroxyproline-rich glycoprotein (TC225721), quaporin (TC223412) and WRKY transcription factor (TC199112), respectively. Relative expression trends of those miRNAs were the same as that predicted by Solexa sequencing and they showed a negative correlation with the expression of the target genes. The results provide molecular evidence for the possible involvement of miRNAs in the process of drought response and/or tolerance in the potato plant.

## Introduction

The potato (*Solanum tuberosum* L.) is one of the most important crops in the world. It is receiving attention for its function as a food for humans and domestic animals as well as for its potential use in future biofuel development [1], but its growth and productivity are severely affected by drought stress. In fact, drought is one of the most frequently occurring environmental stresses that limit agricultural production [2]. Plants respond to drought with an array of biochemical and physiological adaptations mainly dependent on the regulated expression of drought-related genes. To date, a large number of genes that respond to drought stress at the transcriptional level have been identified [3]. The products of these target genes play important roles not only in protecting cells from drought stress, but also in regulating genes for signal transduction in the drought stress response [4]. However, the detailed regulation mechanisms of these genes are incompletely understood.

MicroRNAs (miRNAs) are classes of small, single-stranded, non-coding RNAs found both in plants and animals, which can play important roles in the regulation of gene expression at the post-transcriptional level. They have been considered as mediating gene silencing in response to abiotic stress in model plants [5], [6]. Mature miRNAs are usually 21–24 nucleotides (nt) in length, and can therefore more easily combine with the RNA-induced silencing complex (RISC) to regulate the expression of genes by inhibiting gene translation or degrading targeted mRNAs, depending on the complementarities between miRNA and mRNA [7]. Many miRNAs have been discovered and the majority of their target genes have been identified as encoding transcriptional factors or enzymes that play important roles in many biological processes, including leaf development [8], stem development [9], floral development [10], root development [11], signal transduction [12], phase change [13], and regulation of their own biogenesis [14]. A number of miRNAs also have been identified as being involved in response to drought stress in some plant species such as *Arabidopsis thaliana* [15], rice [16], [17], *Medicago truncatula* [18] and cowpea [19]. Although many plant miRNAs and their targets have been identified, a majority of those studies have focused on some model plant species, such as *Arabidopsis thaliana*, *Oryza sativa*, and *Zea mays* [20]. Since the functions of miRNAs in many other plant species have not been identified, the most challenging problem in understanding the function of plant miRNAs is to identify additional novel miRNAs. While forward genetics, bioinformatics prediction and direct deep-sequencing have been all used for miRNA discovery in plants, there is increasing evidence that deep-sequencing is the most effective method for plant miRNA discovery and expression analysis [16]–[21]. Deep sequencing technology has revolutionized small RNA discovery and a growing number of miRNAs have been identified.

There are several researches on potato miRNAs and their targets identification using computational prediction [22], [23], comparative genome strategy [24], comprehensive bioinformatic analysis of EST data [25] and high-throughput sequencing [26]. However, little has been known about the relationship between identified potato miRNAs and drought stress. Here we report deferentially expressed known and novel miRNAs related to drought stress in the potato using deep sequencing. Moreover we predict the target genes of the miRNAs and identify potential drought-responsive miRNAs and their target genes.

## Results

### Deep Sequencing Results of Potato sRNA Libraries

In order to identify miRNAs related to drought resistance in the potato, sRNA libraries were constructed for both control samples and samples treated with 15% PEG solution. The samples were obtained from the potato tetraploid cultivar ‘Zihuabei’. High-throughput sequencing led to the generation of 17,357,850 and 17,130,641 raw reads from the control and treatment libraries, respectively. There were 16,992,427 clean reads (3,579,840 unique reads) from the control library and 16,824,975 clean reads (4,412,305 unique reads) from the treatment library after initial processing (Table 1). There were 14.62% and 77.33% specific sRNAs in the treatment libraries for total and unique sRNAs, respectively. After initial processing, the high-quality sRNA reads were mapped to the potato genome sequence using SOAP [27]. The numbers of total and unique sequences that matched the genome were 7,243,085/1,715,629 and 7,059,657/2,131,423 in the control and treatment libraries, respectively (Table 1).

**Table 1:** Statistical analysis of sequencing reads for the two sRNAs libraries.

	Total reads	Unique reads
Control		
Raw reads	17,357,850	
Clean reads	16,992,427	3,579,840
Mapped to genome	7,243,085	1,715,629
Mapped to known miRNAs	2,072,796	25,660
Without annotation	6,426,142	2,839,293
Treatment with 15% PEG		
Raw reads	17,130,641	
Clean reads	16,824,975	4,412,305
Mapped to genome	7,059,657	2,131,423
Mapped to known miRNAs	913,806	22,908
Without annotation	7,313,025	3,517,111

**Figure 1:**
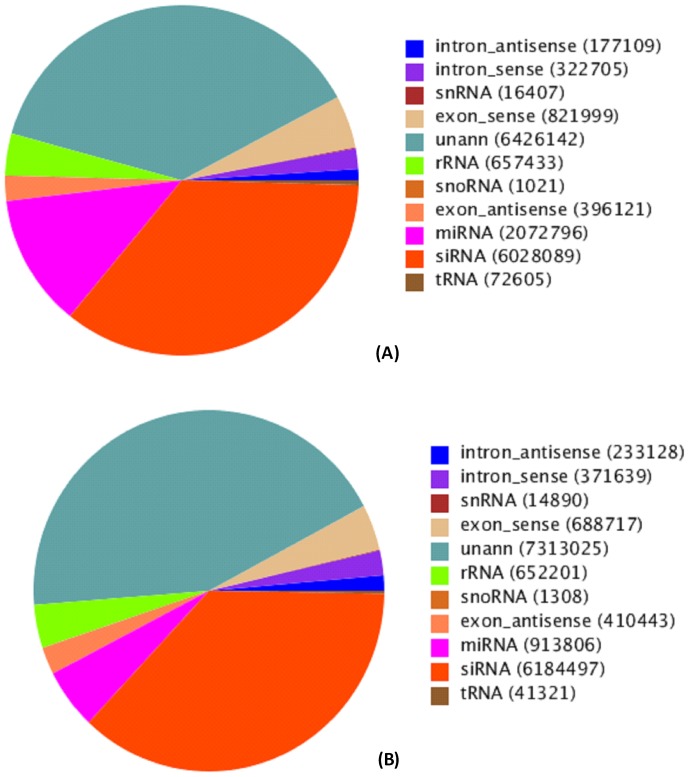
sRNAs annotation and distribution of mRNAs in control (A) and drought treatment (B).

**Figure 2:**
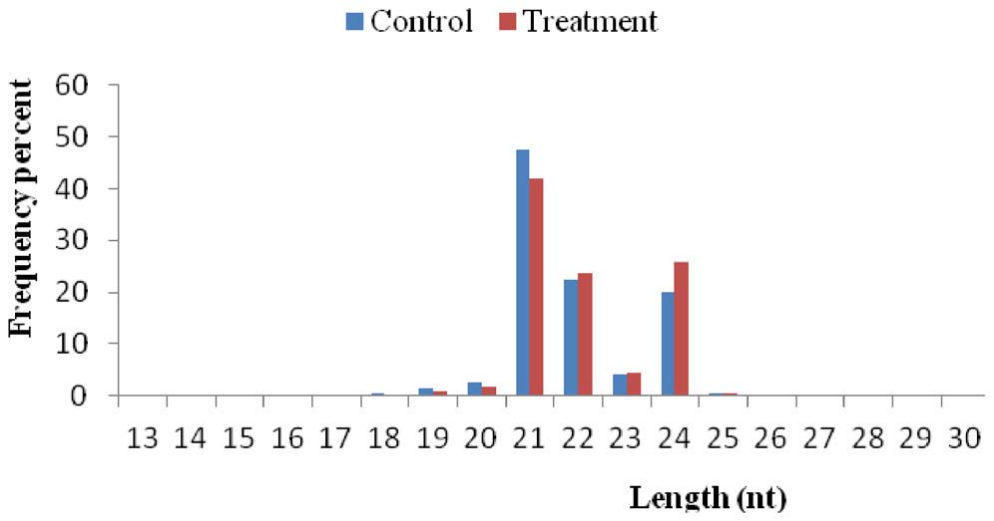
The length distribution of sRNAs in the libraries of control and treatment with 15% PEG in potato.

The tRNA, siRNA, miRNA, intron and exon RNA, snoRNA, rRNA, and snRNA reads were annotated for the total sRNA control and treatment libraries. A total of 2,072,796 and 913,806 known miRNAs were mapped to the control and treatment groups respectively (Fig.1). After removal of contaminant reads, the length distribution of clean reads showed that the majority of the reads were 21 to 24 nt in size, of which the 21 nt class was the most abundant, followed by the 24 nt and 22 nt classes (Fig.2).

### Identification of Known miRNAs in Potato

In order to identify the conserved miRNAs that were obtained from small RNA high-throughput sequencing in potato, we compared this information with the up-dated, known, mature plant miRNAs in the miRBase database. Following Blastn searches and further sequence analysis, a total of 458 and 471 known miRNAs were identified in the control and treatment libraries, respectively. Many of the identified miRNA families have been shown to be conserved in a variety of plant species. Only a few members have been identified in the majority of known miRNA families, whereas some miRNA families contain many potential members which also require further validation based on genomic or EST sequences.

In this study, the read numbers for known miRNAs showed high diversity, ranging from 1 to 656,806 in the control group and from 1 to 300,458 in the treatment group. Among these miRNAs, the miR168 and miR166 families had the most reads in the control and treatment groups, respectively. Additionally, 23 miRNA families were found to have more than ten thousand redundancies in the control group and 17 miRNA families were found to have more than ten thousand redundancies in the treatment group (Table 2). A total of 62 miRNA families had more than one thousand redundancies in the treatment and control groups. The remaining families were infrequently sequenced (less than 1,000 occurrences).

**Table 2:** miRNA families of more than ten thousand redundancies.

Control		Treatment	
miRNA families	Expressional reads	miRNA families	Expressional reads
miR168	656,806	miR166	300,458
miR166	448,342	miR168	172,383
miR5301	205,628	miR157	53,062
miR5076	130,008	miR5301	37,131
miR167	33,888	miR167	19,683
miR171	14,282	miR172	12,936
miR394	19,714	miR5300	11,552
miR156	25,322	miR156	10,339
miR4376	13,123	miR2911	17,603
miR3634	16,791	miR3634	10,033
miR1103	22,171	miR1103	28,052
miR851	24,470	miR169	11,759
miR419	17,009	miR851	15,875
miR5137	20,069	miR419	19,331
miR5052	44,371	miR5052	32,605
miR815	56,091	miR815	32,810
miR1061	34,139	miR1869	42,806
miR1092	26,953		
miR4387	34,678		
miR2870	19,466		
miR3437	20,204		
miR1076	19,564		
miR157	19,237		

### Identification of Novel miRNAs in Potato

A stable hairpin structure is the essential feature for identification of novel miRNAs [28]. In this study, to identify new miRNAs and new members of known miRNA families, we used the unique sRNA sequences from the two libraries to map to the potato genome and predict the secondary structures of a series of sequences surrounding mapped sites. We used the resultant reads to identify novel miRNAs by folding the sequences of potential miRNA precursors using the web-based software Mireap that is publicly available at the website (http://sourceforge.net/projects/mireap/) [29]. Additionally, free energies, and the binding locations of dicer enzymes were used to evaluate these candidate miRNAs. Finally, a total of 674 novel miRNAs in the control samples and 566 novel miRNAs in the drought treatment samples were predicted from high-throughput sequencing results using Mireap software.

### Differential Expression Analysis of Known and Novel miRNAs Under Drought Stress

**Figure 3:**
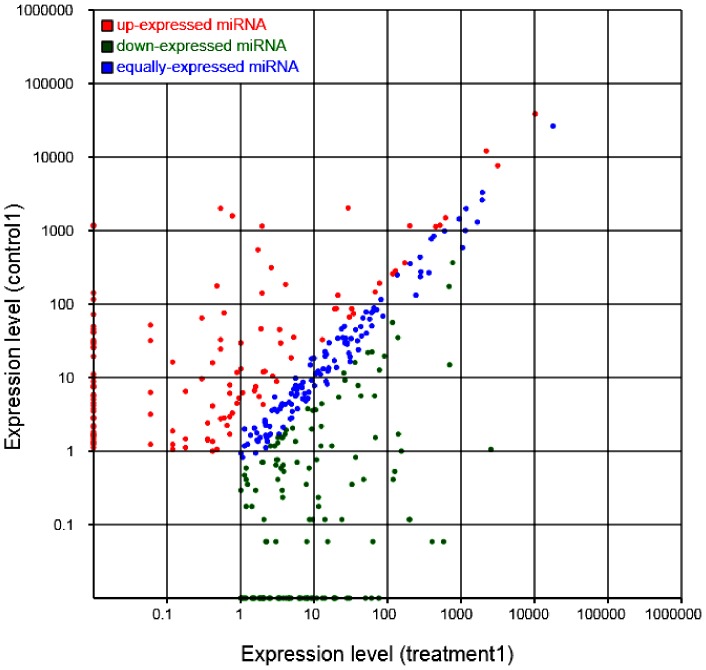
Analysis of known miRNAs differential expression between control and drought treatment. Each point in the figure represents an miRNA. The X axis and Y axis show the expression levels of miRNAs in the control and drought treatment samples, respectively. Colors in the figure: red points represent miRNAs with ratio>2; blue points represent miRNAs with 1/2<ratio≤2; green points represent miRNAs with ratio≤1/2. Ratio = normalized expression in the treatment samples/normalized expression in the control samples.

In order to identify the differentially expressed miRNAs, we compared the expression of the known miRNAs between the control and drought treatment samples by plotting log2-ratios and scatter plots (Fig. 3). Based on the deep sequencing results, the miRNAs with changes in expression levels greater than 2.0-fold in response to drought treatment were selected, which included 100 miRNAs with down-regulation expression pattern and 99 miRNAs with up-regulation expression pattern (Table S1). Based on the miRNAs expression profile, the expression levels of miR3437, miR1076, miR4375, miR3695, miR1037, miR3637, miR1867, miR4235, miR2099, and miR2109 had remarkable differences. The novel miRNAs with changes in expression levels greater than 2.0-fold in response to drought treatment were selected, which included 151 novel miRNAs with down-regulation and 119 novel miRNAs with up-regulation expression patterns (Table S2).

### Target Predictions and Annotation for Differentially Expressed miRNAs

Generally, the targets of plant miRNAs have perfect or near-perfect complementary sites, allowing their identification using bioinformatic prediction methods [30]. We used the online program psRNATarget (http://plantgrn.noble.org/psRNATarget/), Gene Ontology (http://www.Geneontology.org/), and the Kyoto Encyclopedia of Genes and Genomes (http://www.Genome.jp/kegg/) to predict and annotate the target genes. The sixty differentially and highly expressed known and novel miRNAs could each be searched for their potential target genes. All 246 target genes for the known miRNAs and 214 target genes for the novel miRNAs were predicted, and the target ID and target annotation data are shown in (Table S3 and S4). The number of potential targets in the potato varies from zero (stu-miR-662, stu-miR-721 and miR1535) to 16 targets (miR837) per miRNA. Annotation showed that the functions of their target genes were diverse (including involvement in metabolism, cell differentiation, growth and resistance to biotic and abiotic stresses), and their products include transcription factors, enzymes, functional proteins and protein precursor (Table S3 and S4). Based on literatures access [31]–[36] and functional analysis, we determined that 9 target genes of 9 miRNAs are potential drought stress-related genes, and their corresponding 9 miRNAs were therefore considered to be probable drought-related miRNAs. Detailed information about those miRNAs and their target genes is shown in (Table 3).

**Table 3:** Selected potential drought-related miRNAs and their target genes.

miRNAs	Expression pattern	Target ID	Target annotations
miR158	down-regulation	NP440459	Trehalose synthase
miR811	down-regulation	CV431094	MYB transcription factor-*Catharanthus roseus*
miR814	down-regulation	TC225721	Hydroxyproline-rich glycoprotein-*Phaseolus vulgaris*
miR835	down-regulation	TC223412	Aquaporin- *Ricinus communis*
miR1158	down-regulation	TC225596	bZIP family transcription factor-*Arabidopsis thaliana*
miR4398	down –regulation	TC199112	WRKY transcription factor 6-*Solanum tuberosum*
stu-miR-535	down-regulation	TC222240	MYB domain-containing protein
stu-miR-856	up-regulation	TC225708	Mitogen-activated protein kinase 19
stu-miR-860	up-regulation	TC224966	Mitogen-activated protein kinase

### RT-qPCR Analysis of Drought-Responsive miRNAs and their Target Genes

**Figure 4:**
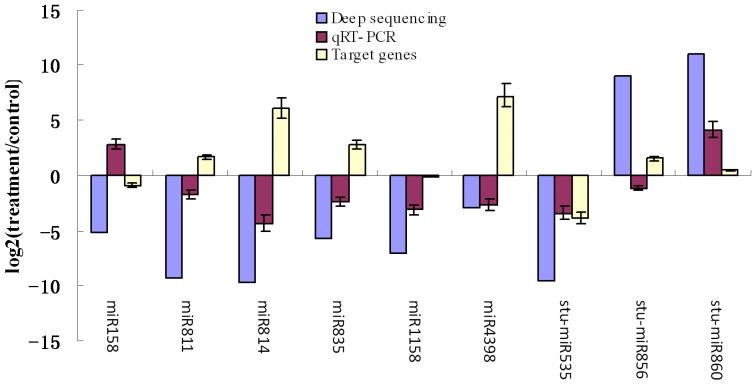
Verification of the selected miRNAs and their potential target genes related to drought stress from deep sequencing by RT-qPCR. Relative expression levels of the selected 9 miRNAs (miR158, miR811, miR814, miR835, miR1158, miR4398, stu-miR535, stu-miR856, stu-miR860) and their potential target genes (NP440459, CV431094, TC225721, TC223412, TC225596, TC199112, TC222240, TC225708, TC224966) were measured by RT-qPCR. The *ef1a* gene was used as an internal control. The expression level is expressed as the mean of relative fold changes of triplicate biological replicates and the vertical bars represent standard derivation of the mean (n = 3).

To confirm and validate the data obtained from the high-throughput sequencing and computational prediction, real-time quantitative PCR (RT-qPCR) analysis was carried out to validate the expression patterns of the 9 drought-responsive miRNAs and their predicted target genes (Fig.4). Comparison of the miRNA from Solexa sequencing and RT-qPCR analyses revealed that the relative expression trends of 7 of the 9 miRNAs in response to the drought treatment were the same as that predicted by Solexa sequencing. These included miR811, miR814, miR835, miR1158, miR4398 and stu-miR535 (which showed down-regulation under drought stress), and stu-miR860 (which showed up-regulation under drought stress). The expression patterns of miR158 and stu-miR-856 showed different expression trends under drought stress conditions between RT-qPCR analysis and Solexa sequencing results. The predicted target genes MYB transcription factor (CV431094), hydroxyproline-rich glycoprotein (TC225721), quaporin (TC223412), WRKY transcription factor (TC199112), mitogen-activated protein kinase 19 (TC225708) and mitogen-activated protein kinase (TC224966) for miR811, miR814, miR835, miR4398, stu-miR-856, stu-miR-860 were up-regulated under drought stress, while target genes trehalose synthase (NP440459), bZIP family transcription factor (TC225596) and MYB domain-containing protein (TC222240) for miR158, miR1158 and stu-miR-535 were down-regulated.

The results from RT-qPCR and Solexa sequencing analysis of the expression patterns of 9 miRNAs and their target genes showed that the relative expression trends of 4 miRNAs were the same for both methods and have a negatively correlated expression pattern with their target genes. Those 4 miRNAs were considered to be potential drought-responsive miRNAs. They include miR811, miR814, miR835, miR4398, and their target genes are MYB transcription factor (CV431094), hydroxyproline-rich glycoprotein (TC225721), quaporin (TC223412) and WRKY transcription factor (TC199112), respectively.

## Discussion

Drought stress is one of the major factors severely limiting plant growth and development, and often results in low crop yields. It is well-known that plants react to drought with an array of gene regulation responses. Many studies have been devoted to transcriptional regulation, in response to high salinity, cold, drought, and ABA treatment in a model plant through cDNA microarray analysis [37], [38]. As a result, a large number of abiotic stress-inducible genes have been identified, contributing to the elucidation of the molecular mechanism of the stress response. Expressional products of induced genes have been reported to function both in the initial stress response and in establishing stress tolerance in plants [39].

miRNAs are a large, new class of small non-coding RNAs, which play important roles in post-transcriptional gene regulation by targeting mRNAs for cleavage or repressing translation. High-throughput sequencing has been used for identification and expressional analysis of miRNAs at the whole genome level in several plant species, including *Arabidopsis* [40], rice [16], [17], *Medicago* [18], cowpea [19], tomato [41], *Populus* [42], wheat [43] and potato [26]. However, most of these studies have focused on miRNAs under non-stressed, normal growth conditions. Only some studies have compared miRNAs under control conditions to those under conditions of abiotic stresses using high-throughput sequencing of plant miRNAs [17]–[19]. Therefore, analysis of the miRNA expression profile under drought stress can contribute to a better understanding of the function of plant miRNAs.

Xie et al. [24] identified 202 potential potato miRNAs using a newly modified comparative genome strategy and predicted 1,094 miRNA targets and found some of targets encoded transcription factors as well as genes that function in stress response, signal transduction, and a variety of other metabolic processes. Zhang et al. [26] characterized small RNAs of potato using high-throughput sequencing and confirmed 28 conserved miRNA families and found 120 potato-specific miRNA families, and predicted miRNA target functions. In this study, we used Solexa sequencing to estimate the genome-wide expression levels of miRNAs in potato plants treated with PEG stress, in order to discover drought responsive miRNAs. A large number of known and novel miRNAs were identified. The results obtained from differential expressional analysis of the miRNAs suggested that miRNAs may play a crucial role in plant response to drought stress. The target genes of the differentially expressed miRNAs obtained from our prediction and annotation can be classified into three clusters: 1) transcription and translation factors (such as zinc finger protein, and MYB domain-containing protein); 2) enzyme and functional protein (such as glucose acyltransferase, glutathione S-transferase, cysteine protease, and peroxidase); and 3) protein or whole genome shot gun sequences with unknown functions in plants (Table S3 and S4). We identified 9 miRNAs as potential targets for drought-related genes. Those potential target genes have a wide variety of predicted functions, which include drought-related transcription factors (MYB, bZIP, WRKY); enzymes (trehalose synthase), and functional proteins (aquaporin). The results from RT-qPCR showed that the relative expression trends of 4 miRNAs (miR811, miR814, miR835 and miR4398) were the same as that predicted by Solexa sequencing, and their accumulation showed a negatively correlated expression pattern with their target genes (MYB transcription factor, hydroxyproline-rich glycoprotein, aquaporin and WRKY transcription factor). MYB transcription factor members have been shown to regulate plant responses to biotic and abiotic stress conditions [33]. WRKY transcription factors are also reported as being involved in plant stress response, which can bind with high affinity to a DNA cis-acting element called the W box ((C/T)TGAC(T/C)) which permits signal transduction to regulate the expression of stress-related genes, resulting in plant stress tolerance. Over-expression of WRKY genes in *Arabidopsis* [34], [35] conferred tolerance to abiotic stresses. Hydroxyproline-rich glycoprotein is a functional protein that can help plants accumulate proline, which is an important osmotic-adjusting material that can help plants adapt to drought and other abiotic stresses [36].

The present results demonstrate that the differentially expressed miRNAs were involved in the biochemical and physiological processes of plant response to drought stress. Further study is necessary to elucidate the functional importance of predicted miRNA-target RNA relationships.

## Materials and Methods

### Plant Material and Drought Treatments

The experiments were carried out with the potato tetraploid cultivar ‘Zihuabei’. The potato tubers (weight: about 150 g) were grown in 3 L pots of vermiculite in a greenhouse, under natural light at (25±2)°C. After sprouting, the plants were watered and fertilized weekly with a complete nutrient solution. When the plants were 30 days old, drought treatments were conducted on the treatment samples by watering 50 ml of 15% PEG solution once a day for 20 days, while plants in the control samples were watered with 50 ml of water daily. Three replicates consisting of 10 plants from each experiment were used for the analysis. Entire plant leaves were then collected from two plants of each replicate and pooled together, and immediately frozen in liquid nitrogen. They were then stored at −80°C for further use.

### Total RNA Extraction and Small RNA Isolation and Sequencing

Total RNA was isolated from the leaves of both the drought treatment and control potato plants using Trizol Reagent (Shanghai Sangon Biotech Co., Ltd., China) according to the manufacturer's instructions. Small RNA libraries were constructed as described by Lagos-Quintana et al. [44]. Briefly, the extracted total RNA was separated by 15% polyacrylamide gel electrophoresis (PAGE) to isolate small RNAs (between 18–30 nt.) These small RNAs were ligated to 5′ adapters (5′-GUUCAGAGUUCUAC AGUCCGACGAUC-3′) and 3′ adapters (5′-UCGUAUGCCGUCUUCUGCUUGU-3′). Subsequently, they were reverse transcribed and amplified using polymerase chain reaction (PCR). The resulting RNA was sequenced by the Beijing Genomics Institute (BGI), using the Solexa technology. All sequence data have been deposited in the Sequence Read Archive (SRA) at the NCBI database (http://www.ncbi.nlm.nih.gov/) under the accession number SRP034924.

### Bioinformatic Identification of Conserved miRNAs

A 50 nt length of raw reads was sequenced. We eliminated some low quality and contaminant reads from the raw reads and obtained the final clean reads using the following steps: (1) eliminating low quality reads; (2) eliminating reads with 5′ primer contaminants; (3) eliminating reads without 3′ primer; (4) eliminating reads without the insert tag; (5) eliminating reads with poly A; (6) eliminating reads shorter than 18 nt; and (7) summarizing the length distribution of the clean reads. Modified sequences from 18 nt to 30 nt long were used for further analysis. The small RNA tags were mapped to the genome using SOAP [27] to analyze their expression and distribution on the genome. We then aligned the small RNA tags to the miRNA precursor/mature miRNA of all plants and animals in the miRBase database [45] to identify the sequence and count of miRNA families (no specific species) in the samples. Only the perfectly matched sequences were considered to be conserved miRNAs.

### Bioinformatic Identification of Novel miRNAs

After removing the conserved miRNA sequences, the remaining small RNA sequences were used to perform Blastn searches in order to obtain novel drought-related miRNAs in the potato. First, rRNA, tRNA, snRNA, and snoRNA, as well as those containing the polyA tail, were removed from the sRNA sequences and the remaining sequences were compared with the sequences in the NCBI GenBank database and Rfam (http://www.sanger.ac.uk/Software/Rfam) database. Then, we aligned small the RNA tags to mRNA exons and introns, to find the degraded fragments of mRNA in the small RNA tags.

Small interfering RNA (siRNA) is a 22–24 nt long, double-strand RNA, each strand of which is 2 nt longer than the other on the 3′ end. According to this structural feature, we aligned tags from the clean reads to each other to find sRNAs meeting the criteria, to identify potential siRNA candidates. Finally, we summarized all alignments and annotations before obtained from the previous steps. This method meant that some small RNA tags could be mapped to more than one category. To make sure that every unique small RNA was mapped to only one annotation, we applied the following priority rule: rRNA etc. (in which GenBank > Rfam) > known miRNA > repeat > exon > intron [46].

We used the web-based software Mireap to predict novel miRNA by exploring the secondary structure, the Dicer cleavage site and the minimum free energy of the unannotated small RNA tags which could be mapped to the genome. Mireap can be accessed from the following link: (http://sourceforge.net/projects/mireap/). The parameters for plants are as follows: (1) minimal miRNA sequence length (18); (2) maximal miRNA sequence length (25); (3) minimal miRNA reference sequence length (20); (4) maximal miRNA reference sequence length (23); (5) maximal copy number of miRNAs on reference (20); (6) maximal free energy allowed for a miRNA precursor (−18 kcal/mol); (7) maximal space between miRNA and miRNA* (300); (8) minimal base pairs of miRNA and miRNA* (16); (9) maximal bulge of miRNA and miRNA* (4); (10) maximal asymmetry of miRNA/miRNA* duplex (4); (11) flank sequence length of miRNA precursor (20).

RNA sequences were considered miRNA candidates only if they fit all of the following criteria: (1) an RNA sequence can fold into an appropriate stem-loop hairpin secondary structure; (2) a mature miRNA sequence site is located in one arm of the hairpin structure; (3) miRNAs had less than 6 mismatches with the opposite miRNA* sequence in the other arm; (4) no loop or break in miRNA* sequences; (5) predicted secondary structures had higher MFEIs, negative MFEs, and 30–70% A+U contents; (6) predicted mature miRNAs had no more than four nucleotide substitutions compared with *Arabidopsis thaliana* and/or rice mature miRNAs. In addition, RNA sequences were considered miRNA candidates only if they fit the criteria for annotation plant miRNAs [28].

### Differential Expression Analysis of Known and Novel miRNAs

In order to identify the differentially expressed miRNAs, we compared the known and novel miRNA expressions between the control and PEG treatment samples by preparing log2-ratios and scatter plots [18]. In the first step, the miRNA expressions in the two samples were normalized to obtain the expression of transcript per million (TPM) on the basis of the normalization formula: normalized expression  =  (actual miRNA count/total count of clean reads) ×1,000,000. In the second step, the fold-change and *p*-values were calculated from the normalized expression. The log2-ratio and scatter plots were then generated. The algorithms are shown as below:

Fold-change formula: fold change  =  log2 (treatment/control)

*P*-value formula:
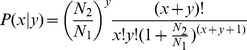


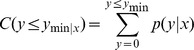


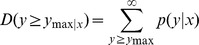



### Prediction and Annotation of Target Genes for Differential Expression of miRNAs

The putative target genes for the thirty most down-regulated and up-regulated differentially expressed known and novel miRNAs were predicted using the plant miRNA target prediction online software psRNATarget (http://plantgrn.noble.org/psRNATarget/) with default parameters [47], which can identify putative targets that may be regulated at post-transcriptional or at translational levels. The parameters are as follows: (1) maximum expectation is less than 3.0; (2) length for complementarity scoring (hspsize) is shorter than 20 nt; (3) target accessibility-allowed maximum energy to unpair the target site (UPE) is shorter than 25; (4) flanking length around target site for target accessibility analysis is 17 bp in upstream and 13 bp in downstream; (5) range of central mismatch leading to translational inhibition is 9–11 nt. Mature miRNA sequences were used as queries to search for potential target mRNAs in the potato database (unigene, DECT Gene Index release 13 on 2010, 04, 16) in order to predict the target genes for differential expression of miRNAs. The Gene Ontology website (http://www.Geneontology.org/) and the Kyoto Encyclopedia of Genes and Genomes (http://www.Genome.jp/kegg/) were used to annotate the function of target genes.

### RT-qPCR Analysis of Drought-related miRNAs and their Predicted Targets

The RT-qPCR technique was used to validate expression profiling of mature miRNAs and their target genes which were obtained from the high-throughput sequencing and bioinformatic prediction for the drought-stressed and control samples. Total RNA was extracted from both drought treated and control plantlets using TRIzol reagent (Invitrogen, USA) following the manufacturer's instructions.

Total RNA (1 µl) was used for synthesizing reverse transcripts using the One Step PrimeScript miRNA cDNA Synthesis Kit (Takara, Japan) in a 20 µl reaction mixture. The reaction was performed at 37°C for 60 minutes and 85°C for 5 seconds following the manufacturer's instructions. The RT-qPCR analysis was performed with SYBR® Premix Ex TaqII (Takara, Japan) to detect and quantify potato miRNAs according to the manufacturer's protocol. The expression levels of 9 obtained miRNAs were analyzed in both the control and treatment samples. The PCR amplification was set at 40 cycles of denaturation at 95°C for 10 seconds, annealing at 60°C for 30 seconds, and extension at 72°C for 1 minute [48]. Reverse primers and Uni-miR qPCR primers provided with the kit, and the miRNA-specific primers were as follows (Table 4).

**Table 4:** RT-qPCR primers for miRNA and their target genes.

miRNAs	miRNA-specific primers sequence	Target ID	Target primers sequence (5′- 3′)
miR158	ACATGCGAGGAGGCTACTGG	NP440459	F: AGCACATGCTCAAACAGCAC
			R: CATACGCTCGCTACTGGACA
miR811	CGTAGTTGATGATGATGGACGG	CV431094	F: GCCAGGCAGAAATTGAGAAG
			R: ATGGAGGGTGACGACAAGAC
miR814	CGGTCGCACTTCATATACTACGAC	TC225721	F: CCACAAAGCACCAAAGGATT
			R: GTGATGGTGGTGGAGGAGAT
miR835	CCGGAGAAGAAGAACTCATAGAAAG	TC223412	F: GGCATGGAGATAGGTGCATT
			R: AACTGCTGGGTTCATTGAGG
miR1158	ACATGCGAGGAGGCTACTGG	TC225596	F: TCACGTGGGCAATGGTAGTA
			R: AGGGGCTAAATCTCCAGGAA
miR4398	GCTTGCTGGAGTGAGAAGACC	TC199112	F: GGGTTAATTCGTGGTCGAGA
			R: ATAATCTTCCGGCGACTTGA
stu-miR-535	CGTGGGTGTGCACAAGTAGAC	TC222240	F: CTTAGGCCCAAAGCAACAAA
			R: GGCCCAATGCTTCCTTTTAT
stu-miR-856	CGGCCTTAATAAGATGGTGAAG	TC225708	F: GCAACAAGATCATCGCAAGA
			R: GAGGCGTGCTCGAAAATATC
stu-miR-860	CGGAGCATTATTGTAGTTGATTTGA	TC224966	F: TATGACCATCACGGGGAAGT
			R: AGCGATTTCACATGGTTTCC

“F” and “R” indicate the direction (forward or reverse) of the primer related to the target gene sequences.

For the RT-qPCR analysis of the miRNA target genes, the 20 ml PCR reaction mixture contained about 100 ng cDNA, 10 ml SYBR Premix Ex Taq II solution (TaKaRa, Japan), and 0.8 µl of each primer. The reactions were gently mixed and incubated at 94°C for 2 minutes, followed by 40 cycles of 94°C for 30 seconds, 60°C for 34 seconds and 72°C for 30 seconds. All sample analysis was performed using 3 biological replicates with 2 technical replicates. The each sample's *ef1a* gene was used as an internal control [49]. These reactions were performed using the ABI3000 (Applied Biosystems 3000 Real-Time PCR) System. The relative level of miRNAs and target genes were detected using the △△Ct method [50], the Ct value was directly compared and transformed into a fold-change difference with the following formula: △△Ct = (△Ct_ treatment_-△Ct_control_), △Ct = Ct_(miRNA/target)_-Ct_ (*ef1a*)_ for each gene. The values within a column of the threshold cycle (Ct) were calculated between control and drought sample groups. The standard deviations of the data were obtained from the three independent experiments with one-way analysis of variance (ANOVA) using statistics software SPSS version 13.0 (SPSS Inc, Chicago, USA).

## Supporting Information

Table S1 **Differentially expressed known miRNAs between control and drought treatment** (miRNAs with fold-change >2.0 or <-2 were listed).
(DOC)

Table S2 **Differentially expressed novel miRNAs between control and drought treatment** (miRNAs with fold-change (log2 >2.0 or<-2 were listed).
(DOC)

Table S3 **Predicted target genes of sixty differentially expressed known miRNAs and their functional annotation.**
(DOC)

Table S4 **Predicted target genes of sixty differentially expressed novel miRNAs and their functional annotation.**
(DOC)
