# Supplementary material for: Identification of Novel and Conserved MicroRNAs Related to Drought Stress in Potato by Deep Sequencing
Source: PLoS One. 2014 Apr 18;9(4):e95489. doi: 10.1371/journal.pone.0095489 (PMC3991665; doi:10.1371/journal.pone.0095489)
Supplement: Table S1 — Differentially expressed known miRNAs between control and drought treatment (miRNAs with fold-change >2.0 or <-2 were listed). (DOC) [file pone.0095489.s001.doc]

**Table S1** Differentially expressed known miRNAs between control and drought treatment (miRNAs with fold-change >2.0 or <-2 were listed below)

| **Expression pattern: up-regulation** | | | **Expression pattern: down-regulation** | | |
| --- | --- | --- | --- | --- | --- |
| miR-name | fold-change | *p*-value | miR-name | fold-change | *p*-value |
| miR161 | 7.09 | 1.58E-79 | miR158 | -5.26 | 6.17E-11 |
| miR 394 | 2.53 | 0 | miR169 | -5.54 | 0 |
| miR472 | 2.11 | 8.54E-168 | miR173 | -10.71 | 0 |
| miR477 | 4.37 | 6.34E-06 | miR319 | -3.89 | 1.68E-64 |
| miR536 | 7.76 | 5.51E-317 | miR395 | -3.39 | 1.49E-41 |
| miR773 | 3.10 | 1.64E-19 | miR414 | -2.55 | 1.38E-10 |
| miR779 | 2.32 | 4.43E-11 | miR437 | -5.60 | 1.22E-52 |
| miR830 | 5.93 | 9.91E-151 | miR443 | -4.14 | 4.55E-09 |
| miR857 | 3.30 | 2.65E-14 | miR445 | -7.21 | 2.61E-08 |
| miR865 | 4.87 | 5.75E-125 | miR446 | -2.20 | 3.51E-184 |
| miR866 | 9.54 | 1.10E-38 | miR529 | -2.96 | 1.91E-10 |
| miR916 | 11.57 | 1.51E-155 | miR538 | -8.92 | 1.36E-25 |
| miR952 | 8.53 | 0 | miR781 | -8.81 | 9.03E-24 |
| miR1026 | 3.73 | 1.02E-158 | miR811 | -9.37 | 1.09E-34 |
| miR1030 | 7.20 | 3.38E-08 | miR814 | -9.66 | 2.89E-42 |
| miR1033 | 12.15 | 2.32E-232 | miR835 | -5.68 | 9.30E-15 |
| miR1037 | 12.82 | 0 | miR837 | -7.27 | 0 |
| miR1038 | 9.12 | 4.05E-29 | miR841 | -3.86 | 3.93E-32 |
| miR1039 | 11.99 | 3.76E-208 | miR859 | -2.86 | 5.76E-12 |
| miR1048 | 5.51 | 5.31E-110 | miR860 | -2.75 | 0 |
| miR1061 | 11.87 | 0 | miR862 | -10.06 | 5.53E-320 |
| miR1073 | 8.55 | 7.47E-20 | miR897 | -2.59 | 3.59E-10 |
| miR1076 | 16.81 | 0 | miR908 | -9.28 | 1.45E-32 |
| miR1088 | 2.09 | 1.28E-07 | miR951 | -10.88 | 3.84E-97 |
| miR1092 | 11.00 | 0 | miR1031 | -3.01 | 2.45E-05 |
| miR1118 | 8.98 | 1.98E-26 | miR1063 | -5.47 | 1.87E-303 |
| miR1122 | 8.82 | 9.72E-24 | miR1079 | -2.22 | 1.36E-07 |
| miR1137 | 11.65 | 1.63E-164 | miR1081 | -2.60 | 5.89E-192 |
| miR1139 | 2.04 | 0 | miR1097 | -2.59 | 3.59E-10 |
| miR1153 | 5.49 | 0 | miR1124 | -11.45 | 3.89E-144 |
| miR1173 | 5.02 | 3.38E-42 | miR1128 | -2.44 | 5.89E-05 |
| miR1222 | 2.74 | 2.40E-93 | miR1136 | -6.74 | 3.47E-06 |
| miR1317 | 7.01 | 2.67E-07 | miR1158 | -7.08 | 1.61E-39 |
| miR1428 | 7.80 | 4.41E-12 | miR1162 | -7.65 | 4.89E-11 |
| miR1446 | 9.60 | 3.54E-40 | miR1172 | -6.53 | 5.81E-156 |
| miR1509 | 7.36 | 4.29E-09 | miR1313 | -7.65 | 4.89E-11 |
| miR1511 | 5.25 | 3.67E-70 | miR1316 | -10.94 | 4.39E-101 |
| miR1515 | 7.51 | 5.45E-10 | miR1426 | -8.57 | 3.92E-20 |
| miR1522 | 2.57 | 1.33E-30 | miR1441 | -6.89 | 8.59E-07 |
| miR1534 | 3.15 | 0 | miR1507 | -4.47 | 1.43E-32 |
| miR1535 | 3.07 | 7.08E-88 | miR1516 | -8.32 | 4.22E-17 |
| miR1856 | 7.36 | 4.29E-09 | miR1523 | -7.47 | 7.98E-10 |
| miR1860 | 8.17 | 2.27E-15 | miR1850 | -10.08 | 3.27E-56 |
| miR1919 | 2.63 | 0 | miR1867 | -12.88 | 0 |
| miR2081 | 8.11 | 9.00E-15 | miR1869 | -11.22 | 0 |
| miR2083 | 6.16 | 0 | miR1873 | -2.21 | 2.33E-20 |
| miR2097 | 9.06 | 5.46E-160 | miR1875 | -3.64 | 5.73E-14 |
| miR2101 | 3.72 | 2.48E-42 | miR1879 | -10.33 | 8.00E-67 |
| miR2105 | 2.54 | 2.09E-16 | miR1881 | -8.83 | 4.49E-24 |
| miR2119 | 12.04 | 1.00E-214 | miR1916 | -2.52 | 2.21E-31 |
| miR2124 | 5.19 | 2.09E-29 | miR2087 | -8.70 | 5.95E-22 |
| miR2604 | 2.23 | 6.30E-17 | miR2089 | -6.84 | 3.13E-227 |
| miR2624 | 6.90 | 0 | miR2099 | -12.55 | 7.42E-308 |
| miR2646 | 11.49 | 1.77E-147 | miR2109 | -12.28 | 5.19E-256 |
| miR2658 | 6.72 | 5.75E-31 | miR2609 | -7.57 | 1.97E-10 |
| miR2664 | 3.37 | 3.96E-05 | miR2610 | -2.34 | 7.18E-88 |
| miR2675 | 8.14 | 4.52E-15 | miR2619 | -11.56 | 2.36E-155 |
| miR2864 | 6.80 | 2.10E-06 | miR2620 | -7.66 | 1.35E-118 |
| miR2868 | 9.53 | 2.20E-38 | miR2635 | -7.70 | 2.43E-11 |
| miR2870 | 9.18 | 0 | miR2636 | -3.99 | 4.35E-15 |
| miR2880 | 6.94 | 5.31 | miR2645 | -7.15 | 5.26E-08 |
| miR2919 | 2.50 | 3.13E-30 | miR2665 | -6.90 | 1.01E-68 |
| miR2922 | 9.98 | 3.92E-52 | miR2667 | -6.20 | 1.52E-41 |
| miR2937 | 3.04 | 1.70E-05 | miR2912 | -6.02 | 2.48E-54 |
| miR2938 | 7.31 | 8.55E-09 | miR3436 | -7.92 | 3.69E-13 |
| miR3435 | 8.43 | 2.33E-18 | miR3442 | -6.33 | 1.03E-45 |
| miR3437 | 16.85 | 0 | miR3454 | -7.88820287 | 0 |
| miR3455 | 9.79 | 7.42E-46 | miR3460 | -3.82 | 6.19E-40 |
| miR3464 | 8.31 | 0 | miR3521 | -9.53 | 1.25E-38 |
| miR3469 | 11.35 | 5.00E-134 | miR3522 | -9.91 | 7.61E-50 |
| miR3509 | 3.98 | 4.28E-08 | miR3633 | -8.17 | 0 |
| miR3512 | 10.12 | 1.63E-57 | miR3636 | -9.82 | 4.07E-47 |
| miR3513 | 4.60 | 4.48E-188 | miR3637 | -13.26 | 0 |
| miR3520 | 2.10 | 3.75E-14 | miR3693 | -5.48 | 2.38E-164 |
| miR3695 | 13.50 | 0 | miR3702 | -8.02 | 7.82E-77 |
| miR3707 | 2.64 | 0 | miR3711 | -3.56 | 1.67E-225 |
| miR4228 | 7.08 | 1.34E-07 | miR3947 | -7.27 | 1.30E-08 |
| miR4345 | 6.99 | 0 | miR3981 | -9.62 | 4.72E-41 |
| miR4348 | 7.72 | 1.74E-11 | miR4221 | -2.06 | 2.22E-07 |
| miR4365 | 7.36 | 4.29E-09 | miR4235 | -12.74 | 0 |
| miR4375 | 13.79 | 0 | miR4240 | -7.70 | 2.43E-11 |
| miR4385 | 3.47 | 1.65E-27 | miR4346 | -2.94 | 7.61E-124 |
| miR4387 | 6.13 | 0 | miR4360 | -8.42 | 2.58E-18 |
| miR4393 | 3.70 | 4.52E-47 | miR4361 | -9.29 | 7.24E-33 |
| miR4402 | 11.31 | 7.65E-130 | miR4366 | -10.73 | 0 |
| miR4404 | 9.20 | 1.29E-30 | miR4372 | -10.85 | 5.09E-95 |
| miR5013 | 9.77 | 1.07E-261 | miR4394 | -2.00 | 2.52E-40 |
| miR5024 | 2.04 | 9.06E-07 | miR4398 | -3.05 | 1.24E-18 |
| miR5029 | 2.24 | 3.28E-07 | miR4413 | -9.69 | 7.16E-43 |
| miR5049 | 5.74 | 2.06E-15 | miR4415 | -2.01 | 5.91E-07 |
| miR5050 | 10.92 | 1.17E-99 | miR5016 | -2.04 | 9.62E-12 |
| miR5071 | 2.37 | 1.84E-07 | miR5065 | -5.22 | 1.21E-10 |
| miR5077 | 2.17 | 6.06E-171 | miR5067 | -8.37 | 1.04E-17 |
| miR5085 | 12.28 | 7.02E-254 | miR5081 | -6.65 | 6.97E-06 |
| miR5140 | 7.41 | 2.16E-09 | miR5138 | -6.65 | 6.97E-06 |
| miR5168 | 2.47 | 9.35E-14 | miR5163 | -7.99 | 9.13E-14 |
| miR5175 | 8.70 | 6.04E-22 | miR5165 | -7.52 | 3.97E-10 |
| miR5176 | 2.75 | 1.20E-07 | miR5170 | -10.45 | 2.79E-72 |
| miR5301 | 2.45 | 0 | miR5184 | -6.81 | 1.72E-06 |
|  |  |  | miR5304 | -6.35 | 0 |
