# Supplementary material for: Identification of Novel and Conserved MicroRNAs Related to Drought Stress in Potato by Deep Sequencing
Source: PLoS One. 2014 Apr 18;9(4):e95489. doi: 10.1371/journal.pone.0095489 (PMC3991665; doi:10.1371/journal.pone.0095489)
Supplement: Table S2 — Differentially expressed novel miRNAs between control and drought treatment (miRNAs with fold-change (log2 >2.0 or<-2 were listed). (DOC) [file pone.0095489.s002.doc]

**Table S2** Differentially expressed novel miRNAs between control and drought treatment (miRNAs with fold-change (log2 >2.0 or<-2 were listed below)

| **Expression pattern: down-regulation** | | | | |
| --- | --- | --- | --- | --- |
| miR-name | Length | Sequence | fold-change | *p*-value |
| stu-miR-1 | 22 | AGGTGTTGATAACTTGGGCATG | -6.80 | 2.11E-06 |
| stu-miR-102 | 21 | TCCAAAGTTTGAGATTGTTAC | -2.79 | 9.34E-17 |
| stu-miR-103 | 21 | AACTGAACTTAAATACACTCC | -2.71 | 2.15E-09 |
| stu-miR-104 | 21 | TCATTTTTCGGGTCGTTACAG | -6.94 | 5.32E-07 |
| stu-miR-105 | 20 | ATTGATTTGCATTTCCCATT | -7.87 | 1.11E-12 |
| stu-miR-107 | 21 | AAGGGAAAAGGTAAAGGTACC | -6.64 | 8.34E-06 |
| stu-miR-108 | 20 | TAGCCAAGGATGACTTGCCT | -9.32 | 2.65E-33 |
| stu-miR-11 | 21 | TTTGAAGAACATGGATTGGGC | -7.14 | 6.74E-08 |
| stu-miR-114 | 23 | ACATGAGTTGTGGTGGGATAATT | -7.31 | 8.56E-09 |
| stu-miR-120 | 22 | TTTGTCAAAGATTTGGAAGCCA | -7.41 | 2.16E-09 |
| stu-miR-125 | 21 | AACGTTTTATAATTTGTTACT | -6.64 | 8.34E-06 |
| stu-miR-127 | 21 | CAGGTAGGAAAAGGGAACAAC | -8.14 | 4.53E-15 |
| stu-miR-131 | 21 | TTTGGAAGAAGGCTTTTTGTA | -6.87 | 1.06E-06 |
| stu-miR-133 | 22 | AGGAAATTTGGGAGAATGGGTA | -7.01 | 2.67E-07 |
| stu-miR-134 | 21 | GAGTATGCACGTCTAGACACC | -2.85 | 8.28E-25 |
| stu-miR-139 | 22 | TGAATTGAGGCAGAGTAGATAC | -10.72 | 1.67E-86 |
| stu-miR-144 | 21 | AAAAAGTAGAGCATATATACC | -11.48 | 5.55E-146 |
| stu-miR-146 | 22 | CTCTTCATTTGATCGGTTGGCC | -6.72 | 4.19E-06 |
| stu-miR-148 | 21 | TGGAGAAGGAGAACACGTGCA | -2.10 | 1.29E-15 |
| stu-miR-152 | 21 | CTTATAAATGATGTTGAACTG | -10.93 | 2.98E-100 |
| stu-miR-153 | 21 | GTTCAAGAAAGTTGTGGGAAA | -12.22 | 5.13E-244 |
| stu-miR-155 | 22 | AAGTCAAGATAGCAACTTTGTA | -11.38 | 5.14E-137 |
| stu-miR-158 | 22 | CAATATGAATGGAATGTAATGC | -6.87 | 1.06E-06 |
| stu-miR-160 | 22 | AAGACATGGTGGAGCAGAGGCA | -10.06 | 4.03E-55 |
| stu-miR-170 | 21 | AGGATGTGTGTCTACTTGTTT | -6.80 | 2.11E-06 |
| stu-miR-172 | 21 | TGCCTGGCTCCCTGTATGCCA | -2.10 | 1.08E-05 |
| stu-miR-175 | 21 | TAACTGCAAAGATTGAAATAA | -7.20 | 3.39E-08 |
| stu-miR-179 | 21 | CATTCTTATCAACATACTGCA | -9.18 | 2.59E-30 |
| stu-miR-180 | 22 | GAGCTTAATGATCGTTTTGATG | -7.87 | 1.11E-12 |
| stu-miR-181 | 22 | TCTAGTGGGAGGGCTAAATCTG | -2.37 | 2.13E-23 |
| stu-miR-193 | 21 | TTATTATTTCAGAAAGTCACT | -7.64 | 6.92E-11 |
| stu-miR-194 | 22 | AGAATTTCGTCAGTGCGGTTAG | -7.55 | 2.74E-10 |
| stu-miR-204 | 21 | TTGATATAATTGGACTTTGGC | -7.25 | 1.70E-08 |
| stu-miR-206 | 21 | GGGCTACTCTCTATTGGCATG | -2.29 | 7.36E-273 |
| stu-miR-209 | 22 | GAGTAGATATAATGAAGATGAT | -7.68 | 3.48E-11 |
| stu-miR-211 | 21 | TTTCAAATATGGTTGGAGATG | -2.58 | 1.76E-49 |
| stu-miR-214 | 21 | AAAGATTGAGACTATCCTATA | -7.46 | 1.09E-09 |
| stu-miR-217 | 22 | AGAAGATCCTAGGAATGATGTA | -7.14 | 6.74E-08 |
| stu-miR-22 | 22 | TTTGATGACTGTATGGAAGGAT | -6.80 | 2.11E-06 |
| stu-miR-229 | 21 | CGAAAAGAGGTAGAAAATTAT | -8.31 | 7.28E-17 |
| stu-miR-231 | 22 | TGATAGTTCAAGTAGAAAAAGG | -7.60 | 1.38E-10 |
| stu-miR-233 | 22 | GGAGGGAACCAGCTACTAGACG | -7.08 | 1.34E-07 |
| stu-miR-242 | 22 | AGGGGCGGCTCAACAGATCTCG | -6.80 | 2.11E-06 |
| stu-miR-243 | 21 | AAAGTTGAACAAGTAGAGACA | -9.61 | 1.78E-40 |
| stu-miR-25 | 22 | AAATGTTGGGAATGAAGGGTGT | -8.91 | 3.12E-25 |
| stu-miR-251 | 21 | AGGATGCGTGGTTAGATTCGG | -2.57 | 2.51E-07 |
| stu-miR-256 | 21 | TTTTTGAAGAGTCTGGGCAAC | -7.55 | 2.74E-10 |
| stu-miR-257 | 22 | GCTCACTGCTCTATCTGTCACC | -7.87 | 1.11E-12 |
| stu-miR-266 | 21 | ATTTCTCGTCAAATCAACTAC | -7.72 | 1.75E-11 |
| stu-miR-267 | 22 | TAATTTCCTAAGATCGTTTTGA | -7.31 | 8.56E-09 |
| stu-miR-273 | 21 | TTGATATAATTGGACTTTGGC | -7.08 | 1.34E-07 |
| stu-miR-276 | 22 | GAGTAGATATAATGAAGATGAT | -6.80 | 2.11E-06 |
| stu-miR-280 | 21 | AAAGATTGAGACTATCCTATA | -7.14 | 6.74E-08 |
| stu-miR-281 | 22 | AGAAGATCCTAGGAATGATGTA | -7.25 | 1.70E-08 |
| stu-miR-283 | 22 | TTTGATGACTGTATGGAAGGAT | -6.87 | 1.06E-06 |
| stu-miR-286 | 21 | CGAAAAGAGGTAGAAAATTAT | -9.93 | 2.44E-50 |
| stu-miR-289 | 22 | TGATAGTTCAAGTAGAAAAAGG | -7.51 | 5.45E-10 |
| stu-miR-298 | 22 | GGAGGGAACCAGCTACTAGACG | -8.57 | 3.76E-20 |
| stu-miR-299 | 22 | AGGGGCGGCTCAACAGATCTCG | -8.31 | 7.28E-17 |
| stu-miR-301 | 21 | AAAGTTGAACAAGTAGAGACA | -10.94 | 1.49E-100 |
| stu-miR-302 | 22 | AAATGTTGGGAATGAAGGGTGT | -8.62 | 9.48E-21 |
| stu-miR-303 | 21 | TTTTTGAAGAGTCTGGGCAAC | -6.72 | 4.19E-06 |
| stu-miR-306 | 21 | TTTCTGGAGATGGCTGGCTAC | -3.06 | 5.27E-55 |
| stu-miR-308 | 22 | GCTCACTGCTCTATCTGTCACC | -7.68 | 3.48E-11 |
| stu-miR-309 | 21 | ATTTCTCGTCAAATCAACTAC | -6.94 | 5.32E-07 |
| stu-miR-31 | 22 | TAATTTCCTAAGATCGTTTTGA | -9.87 | 1.52E-48 |
| stu-miR-313 | 21 | TTGATATAATTGGACTTTGGC | -7.14 | 6.74E-08 |
| stu-miR-317 | 22 | GAGTAGATATAATGAAGATGAT | -6.64 | 8.34E-06 |
| stu-miR-331 | 21 | AAAGATTGAGACTATCCTATA | -6.80 | 2.11E-06 |
| stu-miR-339 | 22 | AGAAGATCCTAGGAATGATGTA | -7.41 | 2.16E-09 |
| stu-miR-340 | 22 | TTTGATGACTGTATGGAAGGAT | -6.94 | 5.32E-07 |
| stu-miR-342 | 21 | CGAAAAGAGGTAGAAAATTAT | -11.21 | 1.79E-121 |
| stu-miR-351 | 23 | AAAAATGATCTTTAGCGACAATT | -6.72 | 4.19E-06 |
| stu-miR-352 | 21 | AAAGTGACATTGGGATGGAGA | -7.84 | 2.22E-12 |
| stu-miR-360 | 21 | AGAAAAGGAGGAACAAAGGTA | -3.40 | 4.44E-38 |
| stu-miR-362 | 21 | CCAATAGGACCACAATTATAT | -7.94 | 2.81E-13 |
| stu-miR-364 | 21 | CGGGATAGTTTAGGTGTGTTT | -8.72 | 3.04E-22 |
| stu-miR-366 | 22 | CAACTAGCTTGTGATTGTCTGT | -6.64 | 8.34E-06 |
| stu-miR-370 | 21 | TGGACAAGTAAAAGTGAACGG | -7.98 | 1.41E-13 |
| stu-miR-374 | 21 | CGTGAGTGGTGGGGTAAGATA | -2.42 | 0 |
| stu-miR-383 | 22 | TGAAGACTTCAACTTGTATACT | -8.68 | 1.20E-21 |
| stu-miR-389 | 20 | CAAGAGGACTTGGTTTATCA | -3.62 | 9.03E-19 |
| stu-miR-4 | 21 | TATTGTAGTTGATTTGATGAG | -10.42 | 1.16E-70 |
| stu-miR-404 | 22 | TCTTGATGTAGGAACGACGAAA | -8.53 | 1.49E-19 |
| stu-miR-416 | 22 | TATTTTATTTTTATCGGGTTCC | -6.80 | 2.11E-06 |
| stu-miR-417 | 22 | CCAATTTCGGGGTCAAAACGGG | -7.08 | 1.34E-07 |
| stu-miR-419 | 23 | AAAGGAAAATACGAAGAAGCATG | -9.62 | 8.94E-41 |
| stu-miR-423 | 21 | AGGTATGACACTCGCGAAGAA | -10.64 | 2.55E-82 |
| stu-miR-427 | 22 | CGAACTTGACACTTCTGGTTAA | -6.94 | 5.32E-07 |
| stu-miR-429 | 21 | AAGCATAGGGAGCTCCTCATC | -6.64 | 8.34E-06 |
| stu-miR-44 | 22 | GGGGCGCGACAGAATGGCGACT | -6.94 | 5.32E-07 |
| stu-miR-447 | 22 | TATAGTTGCAGCAGCTGCAAGT | -2.70 | 1.99E-26 |
| stu-miR-449 | 21 | TCTTGGATTGATTTGATTGTA | -7.01 | 2.67E-07 |
| stu-miR-452 | 22 | CTTTGTATAACTTAAGAAATGG | -6.72 | 4.19E-06 |
| stu-miR-455 | 22 | AATCCAGTGGGCAAAGCTTTTG | -6.80 | 2.11E-06 |
| stu-miR-458 | 22 | ATATGAATGGAATGTAATGCCT | -8.66 | 2.39E-21 |
| stu-miR-46 | 21 | TCAATAGTTCGGGGATGAAAC | -7.72 | 1.75E-11 |
| stu-miR-460 | 21 | GGTTGTAATGTCGGCCTAAGG | -7.01 | 2.67E-07 |
| stu-miR-465 | 21 | AGGTCATCTAGCAGCTTCAAT | -2.47 | 3.69E-252 |
| stu-miR-466 | 22 | TTTTGCTGAGGATGGAAAGACC | -7.01 | 2.67E-07 |
| stu-miR-467 | 22 | AGTTGTGGTATCTGGTTCAAAA | -7.20 | 3.39E-08 |
| stu-miR-473 | 21 | TATTGAACAAGTAGACACATG | -12.20 | 7.84E-240 |
| stu-miR-475 | 21 | TGGTCTATGGTAATGATGGTT | -9.49 | 1.74E-37 |
| stu-miR-476 | 22 | TATCTGAGAAGCATAGGGAATT | -3.30 | 2.18E-62 |
| stu-miR-482 | 21 | TGTGCACAAGTAGACACTTTA | -7.25 | 1.70E-08 |
| stu-miR-485 | 22 | GAGAAGATTGAAGGAGATGCAT | -8.48 | 5.89E-19 |
| stu-miR-495 | 21 | TTGAACAAGTAGACACACATA | -10.19 | 3.34E-60 |
| stu-miR-498 | 22 | CGGGCCTGCTACCGACGCGGAT | -7.80 | 4.41E-12 |
| stu-miR-499 | 23 | TGAAGATAAAGTGCCTGCTCTGA | -4.08 | 1.39E-200 |
| stu-miR-500 | 21 | AAGGGAGTGATAGTTTAGGTA | -6.87 | 1.06E-06 |
| stu-miR-506 | 21 | AGGTAGAGAGATTGTTTCTGA | -6.80 | 2.11E-06 |
| stu-miR-512 | 21 | TGGCAAGCATCCTTGGCGACT | -7.01 | 2.67E-07 |
| stu-miR-513 | 21 | TATATGCTCTAGATTTTGGAC | -11.01 | 6.23E-106 |
| stu-miR-517 | 23 | CACAAGTAGATACTTAAATTTGT | -6.80 | 2.11E-06 |
| stu-miR-528 | 22 | TTTGGATGTTGAAGACTTTTTT | -8.70 | 6.04E-22 |
| stu-miR-53 | 22 | TGAATTATAGGCTTAAACAAAG | -7.01 | 2.67E-07 |
| stu-miR-530 | 21 | GAAGATAGAGAGCACTAATGA | -3.76 | 2.18E-52 |
| stu-miR-535 | 21 | TGGGTGTGCACAAGTAGACAA | -9.54 | 1.11E-38 |
| stu-miR-54 | 21 | GCAGTTCGTAGGATATTGGTG | -6.64 | 8.34E-06 |
| stu-miR-541 | 21 | ACGGGTGAGATGACATTCTTG | -7.68 | 3.48E-11 |
| stu-miR-543 | 21 | TCCAAGCACGAATGTAGAAGT | -6.94 | 5.32E-07 |
| stu-miR-549 | 22 | AGTTCTTGTAGGGTGAGACAAC | -3.28 | 3.15E-21 |
| stu-miR-551 | 22 | TGTGGAAGTTCAGACTGTGTTG | -7.20 | 3.39E-08 |
| stu-miR-556 | 21 | CAGCCAAGGATGACTTGCCGA | -2.44 | 2.85E-07 |
| stu-miR-571 | 23 | TTCGTATTTTCGGTGGCTTCGCT | -7.08 | 1.34E-07 |
| stu-miR-574 | 21 | CAGGCTATGAAAACATTCAGA | -10.96 | 4.79E-102 |
| stu-miR-58 | 21 | TAGAGACGGTATCATTAGAGT | -12.39 | 4.23E-275 |
| stu-miR-580 | 22 | TTGCAAGATTTTAGTGACGACG | -7.80 | 4.41E-12 |
| stu-miR-581 | 23 | AGTTTAATTGCGTTTGGACCATA | -7.87 | 1.11E-12 |
| stu-miR-582 | 21 | TGGACCTGGAGGTAATGGTTT | -7.36 | 4.30E-09 |
| stu-miR-585 | 21 | AGAAATGGAATAGTACTAGTG | -11.74 | 1.13E-174 |
| stu-miR-587 | 22 | AGAGCTACGGAATCAAAACTCA | -7.55 | 2.74E-10 |
| stu-miR-588 | 22 | TGGCATGCGGGTTAGGACGTCG | -8.53 | 1.49E-19 |
| stu-miR-60 | 21 | AAGTAGGAATCAAGGTCAATG | -2.09 | 5.03E-37 |
| stu-miR-66 | 22 | AGCTGCTGACCTATGGATTCCT | -13.26 | 0 |
| stu-miR-67 | 21 | AAAAGTGAAAGTAGAGGATCG | -7.14 | 6.74E-08 |
| stu-miR-7 | 22 | GAGCTTGAGCTCGATTCGAGGG | -7.36 | 4.30E-09 |
| stu-miR-77 | 21 | TCTATTTCAGGAAGTTGGACA | -8.01 | 7.10E-14 |
| stu-miR-80 | 20 | TTGGCATTCTGTCCACCTCC | -8.36 | 1.84E-17 |
| stu-miR-81 | 21 | AGGATATGCAGAAGAAAAATG | -4.22 | 0 |
| stu-miR-82 | 23 | AATAGGGACGGAGGGAGTATATA | -6.64 | 8.34E-06 |
| stu-miR-83 | 22 | ACCTCTGGAGGTCGGTATTGTT | -7.41 | 2.16E-09 |
| stu-miR-86 | 22 | TGTTTAGATGTGCACTCTCAAA | -9.46 | 1.37E-36 |
| stu-miR-87 | 21 | AGGGGCAATTGATAGTTGAGG | -6.94 | 5.32E-07 |
| stu-miR-88 | 21 | GGTTTTAGGGGTAGATGAGGC | -7.31 | 8.56E-09 |
| stu-miR-9 | 21 | AGATATGACATAGGGCTTCAA | -8.55 | 7.47E-20 |
| stu-miR-90 | 21 | ACAATTGATAGTTGACGTGTA | -2.13 | 1.38E-10 |
| stu-miR-93 | 21 | AAGGGTTTCTTACAGAGTTCA | -2.30 | 6.40E-163 |
| stu-miR-95 | 23 | AAACTTAAACTGTTTGATTCTCA | -7.80 | 4.41E-12 |
| stu-miR-97 | 22 | TTCGGTATGGTTGTTGATGGTA | -7.08 | 1.34E-07 |
| stu-miR-98 | 21 | TGGCTGCCAATGAAATGATGC | -7.72 | 1.75E-11 |
| **Expression pattern: up-regulation** | | | | |
| stu-miR-140 | 21 | CGTGAATGATGCGGGAGATAA | 3.38 | 5.67E-51 |
| stu-miR-174 | 21 | GCAGCACCATCAAGATTCACA | 2.516 | 1.98E-13 |
| stu-miR-23 | 21 | GCAGCATCATCAAGATTCACA | 2.82 | 7.58E-31 |
| stu-miR-367 | 22 | AGGGTGAGACTATGCTTATGGA | 2.01 | 3.52E-05 |
| stu-miR-442 | 21 | TTGTTAAGGATTCTAATTGGC | 2.14 | 0 |
| stu-miR-592 | 21 | TTATTGTAGTTGATTTGATGA | 9.84 | 1.01E-47 |
| stu-miR-597 | 21 | AATTCTTGCATGTCGGAATTC | 12.61 | 3.38E-321 |
| stu-miR-599 | 23 | TTCTACAGTAGAAGCGAGCGCCT | 8.54 | 7.89E-20 |
| stu-miR-60 | 21 | AAGTAGGAATCAAGGTCAATG | -2.09 | 5.03E-37 |
| stu-miR-603 | 23 | TGCGATATGAAATTTTTGTGTTA | 7.15 | 5.26E-08 |
| stu-miR-604 | 22 | TGCATAGTAGATCTTACACATC | 6.74 | 3.47E-06 |
| stu-miR-606 | 22 | TGTACCTGCCGAGCTTATGAGG | 6.96 | 4.28E-07 |
| stu-miR-612 | 21 | AGGAACTTAGAAGAAATTGAG | 6.74 | 3.47E-06 |
| stu-miR-613 | 21 | TAGGTGTGCATACTCAAAGTT | 7.42 | 1.60E-09 |
| stu-miR-616 | 22 | TCAATCCATATGGTTGGACGTT | 8.35 | 2.10E-17 |
| stu-miR-622 | 21 | GATTTGGTTCGATTTTCGGTT | 7.47 | 7.98E-10 |
| stu-miR-623 | 22 | TTAATGATATATTTGGATCGGG | 6.65 | 6.98E-06 |
| stu-miR-626 | 21 | GGGTTGTGACCGTTTGAAAGG | 7.78 | 6.03E-12 |
| stu-miR-627 | 22 | AAGAAGACTCAAATACCTTTAA | 6.81 | 1.73E-06 |
| stu-miR-628 | 22 | AAGCTGTGGAGGATTCAAGGCA | 7.70 | 2.43E-11 |
| stu-miR-629 | 22 | GTTTGCATATGTCAGGAGCTTT | 10.62 | 2.24E-81 |
| stu-miR-630 | 22 | TGAACTCTCTCCCTCAATGGCT | 9.40 | 2.72E-35 |
| stu-miR-632 | 23 | ATACATGTGAATCTACTTGAATA | 6.74 | 3.47E-06 |
| stu-miR-633 | 22 | ATGACGGAAGCATAGAGAGCAC | 8.27 | 1.71E-16 |
| stu-miR-634 | 21 | AGCTCCTTTCAGGCCAAGACC | 6.81 | 1.73E-06 |
| stu-miR-640 | 21 | ATGTGACACATTTCGGATTTC | 7.89 | 7.42E-13 |
| stu-miR-641 | 21 | TGTTTAGATGTGCACTCTCAA | 7.92 | 3.69E-13 |
| stu-miR-643 | 21 | TTTGTTGATGGTCATCTATTC | 10.38 | 6.04E-69 |
| stu-miR-645 | 22 | CGAGTGGCTGAGGGGGTTCAAT | 6.74 | 3.47E-06 |
| stu-miR-646 | 23 | CAGATTGCAATTTGGATTTCTGG | 7.61 | 9.83E-11 |
| stu-miR-654 | 21 | ACACAATCAAGAAGAGGTTTT | 7.65 | 4.89E-11 |
| stu-miR-660 | 24 | AGTGAATGAAGCGGGAGTACAAGT | 8.29 | 8.49E-17 |
| stu-miR-662 | 21 | ACACAGCTGACGACTCGTTGA | 10.37 | 1.21E-68 |
| stu-miR-664 | 21 | GAACAACTATTGTTGGACGGA | 9.40 | 2.72E-35 |
| stu-miR-668 | 22 | AACGAACGGTTTGATGCGTTGG | 11.53 | 2.54E-152 |
| stu-miR-672 | 22 | TATTTGGACCAAGTATTTGATG | 8.92 | 1.37E-25 |
| stu-miR-673 | 21 | CACGTGTTCTCCTTCTCCAAC | 6.89 | 8.59E-07 |
| stu-miR-674 | 21 | TTCCAAAGCTGCAGAAATGAG | 8.45 | 1.29E-18 |
| stu-miR-677 | 22 | TAGGTCTGAGATATATATTGCT | 7.37 | 3.23E-09 |
| stu-miR-680 | 21 | TTCCACAGCTTTCTTGAACTT | 11.01 | 1.25E-105 |
| stu-miR-683 | 22 | TGAAGACTAGGCTTGAAGTATT | 8.37 | 1.05E-17 |
| stu-miR-684 | 21 | GTGTGTGGCTGAAGTTGTGCA | 9.92 | 1.89E-50 |
| stu-miR-686 | 22 | AGACATAAACATACTTGGTTCA | 7.74 | 1.21E-11 |
| stu-miR-694 | 22 | GTTAGTTGAGGCCAAGTTTGAG | 7.37 | 3.23E-09 |
| stu-miR-695 | 22 | AGAAGATCGAAGGAAAACGGAG | 8.52 | 1.59E-19 |
| stu-miR-700 | 21 | CAATTTCAGTGATTGTGGGAC | 9.58 | 7.71E-40 |
| stu-miR-715 | 23 | ATCAGCTAACTGTATTCGTCTTA | 7.27 | 1.30E-08 |
| stu-miR-717 | 22 | TGGTAGAACACTTTGGGGACGT | 7.27 | 1.30E-08 |
| stu-miR-719 | 22 | TAGGGACTACTAGGGTTTAGGA | 7.99 | 9.14E-14 |
| stu-miR-721 | 21 | AGATCATGTGGCAGCATCACC | 10.51 | 1.29E-75 |
| stu-miR-724 | 21 | AGGTAGAAAGACTGTTTCCAA | 8.92 | 1.37E-25 |
| stu-miR-727 | 22 | TTATACAGAGAAACCGCTGTCG | 8.57 | 3.93E-20 |
| stu-miR-732 | 22 | TGATGCAGGTCACTAGGAATGA | 7.96 | 1.84E-13 |
| stu-miR-733 | 22 | GGCAGACTACATTTTCTTTGAA | 10.18 | 3.74E-60 |
| stu-miR-737 | 22 | GAACCACAGTGAGGTCGTTGTA | 8.09 | 1.13E-14 |
| stu-miR-742 | 22 | AGGCTTAGATATGACATAGGGC | 9.33 | 8.92E-34 |
| stu-miR-747 | 21 | CAAATGGAATCTGGCAACTTT | 6.65 | 6.98E-06 |
| stu-miR-750 | 22 | AGGGTTCAATGTAGTCATCAAA | 7.47 | 7.98E-10 |
| stu-miR-752 | 22 | CAATTAAAAGTGGACGAAGGTA | 6.89 | 8.59E-07 |
| stu-miR-753 | 22 | AGTGGACAACTATTATTGGACA | 6.89 | 8.59E-07 |
| stu-miR-754 | 21 | GTAGATCTTCATATGTTGGGA | 7.65 | 4.89E-11 |
| stu-miR-755 | 22 | CATTTATGACTTGCTAGGACTG | 8.32 | 4.22E-17 |
| stu-miR-759 | 23 | AAAATCAAATTTTGATCCGTCTG | 7.96 | 1.84E-13 |
| stu-miR-762 | 22 | AAGGGTGATGGTGGGTATTGAA | 7.89 | 7.42E-13 |
| stu-miR-763 | 22 | ACATTTGTGGAAGTTGGTAGTC | 6.74 | 3.47E-06 |
| stu-miR-764 | 22 | TGCGTTTACTCAGAATGTTGAC | 7.21 | 2.62E-08 |
| stu-miR-766 | 23 | ATTCGGATTCACATTAGAAGGTT | 7.89 | 7.42E-13 |
| stu-miR-767 | 21 | GGACAATTATTGTTGGACGGA | 7.92 | 3.69E-13 |
| stu-miR-77 | 21 | TCTATTTCAGGAAGTTGGACA | -8.01 | 7.10E-14 |
| stu-miR-770 | 22 | TGGCACTTAACATACTTCTGTT | 7.32 | 6.48E-09 |
| stu-miR-774 | 21 | GGAGCATCATCAAGATTCACA | 7.57 | 1.98E-10 |
| stu-miR-776 | 21 | CCATTTGGTCTTTGGAAAATA | 7.47 | 7.98E-10 |
| stu-miR-777 | 23 | AAGTAGAGCTGTCAATATGGGCT | 6.65 | 6.98E-06 |
| stu-miR-780 | 22 | AGTTGTTGGTTGTGGGTGGGGT | 6.74 | 3.47E-06 |
| stu-miR-783 | 22 | TTTTGGGTTGGTAGAAAGGTGG | 9.35 | 4.44E-34 |
| stu-miR-786 | 22 | TAAAGACCCAAGATACTTGAAA | 7.37 | 3.23E-09 |
| stu-miR-787 | 21 | GTAAGATCTGCGTACATTCTA | 7.89 | 7.42E-13 |
| stu-miR-788 | 22 | TGGGAGCAATAAGATGATTGGG | 7.74 | 1.21E-11 |
| stu-miR-790 | 23 | ATCATGACAATTAAAAGTGGACA | 7.03 | 2.13E-07 |
| stu-miR-792 | 22 | TAATCGGTGTCGAATTAGGCTG | 9.61 | 9.49E-41 |
| stu-miR-793 | 22 | AGGTAGTACTGAGTAGGATTTT | 7.47 | 7.98E-10 |
| stu-miR-798 | 21 | GGACAAGTAAAGATGGACGGA | 7.42 | 1.60E-09 |
| stu-miR-799 | 22 | TTTGTCCTAGAATTTGCAAGTA | 7.37 | 3.23E-09 |
| stu-miR-800 | 23 | AGTAACTCTGTCCACCAAGGCTA | 7.21 | 2.62E-08 |
| stu-miR-801 | 22 | AAGGTACCTAACAATTTTCTAA | 7.15 | 5.26E-08 |
| stu-miR-803 | 23 | ACTATTGACTTTGCATACTTCTT | 7.37 | 3.23E-09 |
| stu-miR-805 | 20 | GTTATTCTATTCCACCTCTT | 6.89 | 8.59E-07 |
| stu-miR-807 | 21 | AGAAGTAGGAAGGCGACCCAA | 7.27 | 1.30E-08 |
| stu-miR-810 | 21 | GTCCAAGAAAGCTGTGGGAAA | 12.62 | 9.88E-323 |
| stu-miR-820 | 22 | AGTCTTGTAGAGGCCCATCTTA | 7.42 | 1.60E-09 |
| stu-miR-824 | 22 | ATGTGGAGATTGTTCCTGATCG | 7.61 | 9.83E-11 |
| stu-miR-829 | 22 | TGTTAGAAATGGTAGACTTCGA | 6.81 | 1.73E-06 |
| stu-miR-833 | 22 | TCCAAAATTGATAGGAGATCGA | 6.96 | 4.28E-07 |
| stu-miR-834 | 22 | TTCTGAAGTAGAGTGGATATTA | 6.74 | 3.47E-06 |
| stu-miR-837 | 23 | AGTGAATGATGCGGTAGACTGAT | 7.37 | 3.23E-09 |
| stu-miR-842 | 21 | TGAAGAGATTGATAGCACGTG | 13.78 | 0 |
| stu-miR-845 | 22 | TCACAAGTTCTTCTGCATGGCG | 6.89 | 8.59E-07 |
| stu-miR-848 | 22 | TGGACACGAAATATGGGACGCA | 12.59 | 1.19E-316 |
| stu-miR-85 | 21 | TCTGGACAAAGATGAGAAGGA | 2.01 | 0 |
| stu-miR-851 | 22 | TTTGAAGAACATGGATTGGGCT | 7.03 | 2.13E-07 |
| stu-miR-852 | 22 | AAGGAGATGAGCAATTCACTAT | 7.09 | 1.06E-07 |
| stu-miR-853 | 21 | AAGATGATTCATATGTGTTAA | 8.09 | 1.13E-14 |
| stu-miR-856 | 22 | CGGCCTTAATAAGATGGTGAAG | 8.96 | 3.39E-26 |
| stu-miR-860 | 22 | AGCATTATTGTAGTTGATTTGA | 11.02 | 3.80E-107 |
| stu-miR-861 | 22 | TCTCCTTGAAGACACACTCTAG | 10.97 | 3.32E-103 |
| stu-miR-864 | 22 | AAGGTAATCTTAGTCTTGGATA | 10.61 | 9.05E-81 |
| stu-miR-866 | 22 | TCTAAGCCTGAGATTACCTTTG | 10.87 | 1.55E-96 |
| stu-miR-873 | 22 | CCGGAGCTAACCGCGCTGACGG | 8.37 | 1.05E-17 |
| stu-miR-874 | 22 | TCATGTGATGGTTGTCGGTTAG | 7.85 | 1.49E-12 |
| stu-miR-880 | 22 | CAATCTTGTTGGTAATTTGGAT | 7.78 | 6.03E-12 |
| stu-miR-886 | 22 | AGTGTGGTGTGGAGGCATGAGT | 8.78 | 3.65E-23 |
| stu-miR-891 | 21 | AACATTTGCATATCCGTTCTA | 8.47 | 6.41E-19 |
| stu-miR-899 | 21 | TTTTTGTTAGACTATTTTATA | 9.85 | 5.02E-48 |
| stu-miR-901 | 22 | TGGTATTGTACTAAGACGGTTT | 7.27 | 1.30E-08 |
| stu-miR-902 | 21 | TGGAAGAGATGATTGGAGAAA | 7.32 | 6.48E-09 |
| stu-miR-909 | 21 | ACTGCATACTTGTTACCTGAA | 7.89 | 7.42E-13 |
| stu-miR-910 | 21 | GACGGATAGAAAAGCGACGGA | 7.57 | 1.98E-10 |
| stu-miR-911 | 22 | AGCTACTGTGGCTCAGCGGTTG | 7.37 | 3.23E-09 |
| stu-miR-918 | 21 | GAACAAGTATTGTTGGACATT | 9.41 | 1.35E-35 |
| stu-miR-920 | 21 | TGAAAGTTGGCAGCCACATTT | 7.74 | 1.21E-11 |
