# Supplementary material for: Identification of Novel and Conserved MicroRNAs Related to Drought Stress in Potato by Deep Sequencing
Source: PLoS One. 2014 Apr 18;9(4):e95489. doi: 10.1371/journal.pone.0095489 (PMC3991665; doi:10.1371/journal.pone.0095489)
Supplement: Table S3 — Predicted target genes of sixty differentially expressed known miRNAs and their functional annotation. (DOC) [file pone.0095489.s003.doc]

**Table S3 Predicted target genes of sixty differentially expressed known miRNAs and their functional annotation**

| **Expression pattern: down-regulation** | | |
| --- | --- | --- |
| miRNAs | Target ID | Target annotations |
| miR1063 | TC204937  CN514145  AM908173  TC203028  CV286408  TC211500  TC225431 | Catechol O-methyltransferase  Hydroxyproline-rich glycoprotein  40S 40S ribosomal protein S17-like protein  Patatin-2-Kuras 3 precursor  Whole genome shotgun sequence  Whole genome shotgun sequence  EMB2410 (EMBRYO DEFECTIVE 2410) |
| miR1124 | [CN514729](http://plantgrn.noble.org/psRNATarget/getseq.do?sessionid=1362323945595991&source=target&seqID=CN514729)  [TC218546](http://plantgrn.noble.org/psRNATarget/getseq.do?sessionid=1362323945595991&source=target&seqID=TC218546)  [TC198051](http://plantgrn.noble.org/psRNATarget/getseq.do?sessionid=1362323945595991&source=target&seqID=TC198051)  [TC222382](http://plantgrn.noble.org/psRNATarget/getseq.do?sessionid=1362323945595991&source=target&seqID=TC222382)  [TC194665](http://plantgrn.noble.org/psRNATarget/getseq.do?sessionid=1362323945595991&source=target&seqID=TC194665) | Predicted protein-*Sclerotinia sclerotiorum* (strain ATCC 18683 / 1980 / Ss-1)  Aspartic protease-like  Aspartic protease-like  Aspartic protease-like  Aspartic protease-like |
| miR1172 | [CV476461](http://plantgrn.noble.org/psRNATarget/getseq.do?sessionid=1362324376212601&source=target&seqID=CV476461)  [TC222414](http://plantgrn.noble.org/psRNATarget/getseq.do?sessionid=1362324376212601&source=target&seqID=TC222414)  [CV499690](http://plantgrn.noble.org/psRNATarget/getseq.do?sessionid=1362324376212601&source=target&seqID=CV499690)  [TC200535](http://plantgrn.noble.org/psRNATarget/getseq.do?sessionid=1362324376212601&source=target&seqID=TC200535)  [TC195086](http://plantgrn.noble.org/psRNATarget/getseq.do?sessionid=1362324376212601&source=target&seqID=TC195086)  [TC207060](http://plantgrn.noble.org/psRNATarget/getseq.do?sessionid=1362324376212601&source=target&seqID=TC207060)  [TC223745](http://plantgrn.noble.org/psRNATarget/getseq.do?sessionid=1362324376212601&source=target&seqID=TC223745) | Chromosome chr7 whole genome shotgun sequence  Chromosome chr14, whole genome shotgun sequence  Chromosome chr4, whole genome shotgun sequence  Chromosome chr14, whole genome shotgun sequence  Aminomethyltransferase, mitochondrial precursor  Aminomethyltransferase, mitochondrial precursor  Chromosome chr3 scaff, whole genome shotgun sequence |
| miR1316 | [TC194915](http://plantgrn.noble.org/psRNATarget/getseq.do?sessionid=1362325454281881&source=target&seqID=TC194915)  [CV492475](http://plantgrn.noble.org/psRNATarget/getseq.do?sessionid=1362325454281881&source=target&seqID=CV492475)  [TC212211](http://plantgrn.noble.org/psRNATarget/getseq.do?sessionid=1362325454281881&source=target&seqID=TC212211)  [TC198954](http://plantgrn.noble.org/psRNATarget/getseq.do?sessionid=1362325454281881&source=target&seqID=TC198954)  [TC216343](http://plantgrn.noble.org/psRNATarget/getseq.do?sessionid=1362325454281881&source=target&seqID=TC216343)  [TC199445](http://plantgrn.noble.org/psRNATarget/getseq.do?sessionid=1362325454281881&source=target&seqID=TC199445)  [BQ516815](http://plantgrn.noble.org/psRNATarget/getseq.do?sessionid=1362325454281881&source=target&seqID=BQ516815) | Derlin-2.2  Protein yippee-like  Chromosome chr10, whole genome shotgun sequence  Chromosome chr10, whole genome shotgun sequence  60s acidic ribosomal protein-like protein  2-oxoglutarate dehydrogenase E2 subunit  Photosystem I reaction center subunit X psaK |
| miR1507 | [TC209328](http://plantgrn.noble.org/psRNATarget/getseq.do?sessionid=1362326256928570&source=target&seqID=TC209328)  [TC226481](http://plantgrn.noble.org/psRNATarget/getseq.do?sessionid=1362326256928570&source=target&seqID=TC226481)  [NP919978](http://plantgrn.noble.org/psRNATarget/getseq.do?sessionid=1362326256928570&source=target&seqID=NP919978)  [TC216998](http://plantgrn.noble.org/psRNATarget/getseq.do?sessionid=1362326256928570&source=target&seqID=TC216998) | Truncated RB  Blight resistance protein SH20  Blight resistance protein SH10  Blight resistance protein SH20 |
| miR1850 | [TC211456](http://plantgrn.noble.org/psRNATarget/getseq.do?sessionid=1362326955842168&source=target&seqID=TC211456)  [CV469428](http://plantgrn.noble.org/psRNATarget/getseq.do?sessionid=1362326955842168&source=target&seqID=CV469428)  [DV627604](http://plantgrn.noble.org/psRNATarget/getseq.do?sessionid=1362326955842168&source=target&seqID=DV627604)  [TC200783](http://plantgrn.noble.org/psRNATarget/getseq.do?sessionid=1362326955842168&source=target&seqID=TC200783)  [TC213387](http://plantgrn.noble.org/psRNATarget/getseq.do?sessionid=1362326955842168&source=target&seqID=TC213387)  [BG350630](http://plantgrn.noble.org/psRNATarget/getseq.do?sessionid=1362326955842168&source=target&seqID=BG350630)  [TC220303](http://plantgrn.noble.org/psRNATarget/getseq.do?sessionid=1362326955842168&source=target&seqID=TC220303)  [DV626021](http://plantgrn.noble.org/psRNATarget/getseq.do?sessionid=1362326955842168&source=target&seqID=DV626021)  [TC226099](http://plantgrn.noble.org/psRNATarget/getseq.do?sessionid=1362326955842168&source=target&seqID=TC226099) | NADH-quinone oxidoreductase subunit J  DNAJ heat shock N-terminal domain-containing protein-like  Probable serine protease inhibitor 6 precursor  Blue copper-like protein  4,5-DOPA dioxygenase extradiol  Chromosome chr, whole genome shotgun sequence  Cell wall protein  Os05g0374700 protein  4,5-DOPA dioxygenase extradiol |
| miR1867 | [CK717603](http://plantgrn.noble.org/psRNATarget/getseq.do?sessionid=1362327474424727&source=target&seqID=CK717603)  [CV474504](http://plantgrn.noble.org/psRNATarget/getseq.do?sessionid=1362327474424727&source=target&seqID=CV474504) [AM909306](http://plantgrn.noble.org/psRNATarget/getseq.do?sessionid=1362327474424727&source=target&seqID=AM909306)  [EG563084](http://plantgrn.noble.org/psRNATarget/getseq.do?sessionid=1362327474424727&source=target&seqID=EG563084)  [TC212287](http://plantgrn.noble.org/psRNATarget/getseq.do?sessionid=1362327474424727&source=target&seqID=TC212287)  [CV500953](http://plantgrn.noble.org/psRNATarget/getseq.do?sessionid=1362327474424727&source=target&seqID=CV500953)  [DN907333](http://plantgrn.noble.org/psRNATarget/getseq.do?sessionid=1362327474424727&source=target&seqID=DN907333) | Spermidine synthase  Similar to UniRef100_UPI0000ECA960 Cluster: LS14B_HUMAN Isoform 2  Small zinc finger-like protein  Small zinc finger-like protein  Small zinc finger-like protein  Inorganic pyrophosphatase  ABC transporter, permease protein |
| miR1869 | [TC202940](http://plantgrn.noble.org/psRNATarget/getseq.do?sessionid=1362328173336358&source=target&seqID=TC202940) | Pyruvate dehydrogenase E1 beta subunit isoform 2 |
| miR1879 | [TC195180](http://plantgrn.noble.org/psRNATarget/getseq.do?sessionid=1362328226054376&source=target&seqID=TC195180)  [TC209481](http://plantgrn.noble.org/psRNATarget/getseq.do?sessionid=1362328226054376&source=target&seqID=TC209481)  [AW096896](http://plantgrn.noble.org/psRNATarget/getseq.do?sessionid=1362328226054376&source=target&seqID=AW096896)  [TC224791](http://plantgrn.noble.org/psRNATarget/getseq.do?sessionid=1362328226054376&source=target&seqID=TC224791)  [TC201547](http://plantgrn.noble.org/psRNATarget/getseq.do?sessionid=1362328226054376&source=target&seqID=TC201547) | Fiber protein Fb19  Methionine rich arabinogalactan  Methionine rich arabinogalactan  Methionine rich arabinogalactan  F8L10.9 protein |
| miR2089 | [TC204762](http://plantgrn.noble.org/psRNATarget/getseq.do?sessionid=1362328530534630&source=target&seqID=TC204762)  [BE922282](http://plantgrn.noble.org/psRNATarget/getseq.do?sessionid=1362328530534630&source=target&seqID=BE922282)  [TC202298](http://plantgrn.noble.org/psRNATarget/getseq.do?sessionid=1362328530534630&source=target&seqID=TC202298) | Molybdopterin synthase sulphurylase  Nongradient byssal  Whole genome shotgun sequence |
| miR2099 | [TC203272](http://plantgrn.noble.org/psRNATarget/getseq.do?sessionid=1362328568012867&source=target&seqID=TC203272) | GTPase-like |
| miR2610 | [CV493858](http://plantgrn.noble.org/psRNATarget/getseq.do?sessionid=1362328837457762&source=target&seqID=CV493858)  [CV504353](http://plantgrn.noble.org/psRNATarget/getseq.do?sessionid=1362328837457762&source=target&seqID=CV504353) | Actin depolymerizing factor 6  Actin depolymerizing factor 6 |
| miR2619 | [CV502801](http://plantgrn.noble.org/psRNATarget/getseq.do?sessionid=1362328875610538&source=target&seqID=CV502801) | Dihydrofolate reductase-thymidylate synthase |
| miR2665 | [TC226204](http://plantgrn.noble.org/psRNATarget/getseq.do?sessionid=1362329150632731&source=target&seqID=TC226204)  [TC205585](http://plantgrn.noble.org/psRNATarget/getseq.do?sessionid=1362329150632731&source=target&seqID=TC205585)  [EG016084](http://plantgrn.noble.org/psRNATarget/getseq.do?sessionid=1362329150632731&source=target&seqID=EG016084) | Rapid alkalinization factor 1  Valyl-tRNA synthetase  Calcineurin B-like interacting protein kinase |
| miR2912 | [CV502990](http://plantgrn.noble.org/psRNATarget/getseq.do?sessionid=1362329468739148&source=target&seqID=CV502990) | Sodium/hydrogen exchanger |
| miR3442 | [TC199312](http://plantgrn.noble.org/psRNATarget/getseq.do?sessionid=1362357386182482&source=target&seqID=TC199312)  [BG350866](http://plantgrn.noble.org/psRNATarget/getseq.do?sessionid=1362357386182482&source=target&seqID=BG350866) | Ubiquitin carrier protein  Probable malate:quinone oxidoreductase |
| miR3522 | [DN942028](http://plantgrn.noble.org/psRNATarget/getseq.do?sessionid=1362357515350543&source=target&seqID=DN942028)  [BG096599](http://plantgrn.noble.org/psRNATarget/getseq.do?sessionid=1362357515350543&source=target&seqID=BG096599)  [TC219161](http://plantgrn.noble.org/psRNATarget/getseq.do?sessionid=1362357515350543&source=target&seqID=TC219161) | Triosephosphate isomerase  Xanthine/uracil permease family protein  Xanthine/uracil permease family protein |
| miR3633 | [CK864116](http://plantgrn.noble.org/psRNATarget/getseq.do?sessionid=1362357865363434&source=target&seqID=CK864116)  [DN906150](http://plantgrn.noble.org/psRNATarget/getseq.do?sessionid=1362357865363434&source=target&seqID=DN906150)  [TC215600](http://plantgrn.noble.org/psRNATarget/getseq.do?sessionid=1362357865363434&source=target&seqID=TC215600)  [TC202894](http://plantgrn.noble.org/psRNATarget/getseq.do?sessionid=1362357865363434&source=target&seqID=TC202894)  [TC220215](http://plantgrn.noble.org/psRNATarget/getseq.do?sessionid=1362357865363434&source=target&seqID=TC220215)  [TC209843](http://plantgrn.noble.org/psRNATarget/getseq.do?sessionid=1362357865363434&source=target&seqID=TC209843)  [TC226304](http://plantgrn.noble.org/psRNATarget/getseq.do?sessionid=1362357865363434&source=target&seqID=TC226304)  [TC200888](http://plantgrn.noble.org/psRNATarget/getseq.do?sessionid=1362357865363434&source=target&seqID=TC200888)  [AM909536](http://plantgrn.noble.org/psRNATarget/getseq.do?sessionid=1362357865363434&source=target&seqID=AM909536)  [DN942162](http://plantgrn.noble.org/psRNATarget/getseq.do?sessionid=1362357865363434&source=target&seqID=DN942162) | At1g52565  Double WRKY type transfactor  G-box binding protein  G-box binding protein  At1g52565  Os02g0201300 protein  Potato late blight resistance protein R3a  Viroid RNA-binding protein  60S ribosomal protein L13  Sensor protein |
| miR3636 | [TC194985](http://plantgrn.noble.org/psRNATarget/getseq.do?sessionid=1362357904196167&source=target&seqID=TC194985) | Inosine-5'-phosphate dehydrogenase |
| miR3693 | [TC212000](http://plantgrn.noble.org/psRNATarget/getseq.do?sessionid=1362358544477368&source=target&seqID=TC212000) | OBP33pep like protein |
| miR3702 | [DN905960](http://plantgrn.noble.org/psRNATarget/getseq.do?sessionid=1362358728181399&source=target&seqID=DN905960)  [EL732282](http://plantgrn.noble.org/psRNATarget/getseq.do?sessionid=1362358728181399&source=target&seqID=EL732282) | Expressed protein  Predicted protein |
| miR395 | [EG010436](http://plantgrn.noble.org/psRNATarget/getseq.do?sessionid=1362359014168174&source=target&seqID=EG010436)  [TC198548](http://plantgrn.noble.org/psRNATarget/getseq.do?sessionid=1362359014168174&source=target&seqID=TC198548)  [TC203808](http://plantgrn.noble.org/psRNATarget/getseq.do?sessionid=1362359014168174&source=target&seqID=TC203808)  [TC197035](http://plantgrn.noble.org/psRNATarget/getseq.do?sessionid=1362359014168174&source=target&seqID=TC197035)  [TC198237](http://plantgrn.noble.org/psRNATarget/getseq.do?sessionid=1362359014168174&source=target&seqID=TC198237)  [TC198305](http://plantgrn.noble.org/psRNATarget/getseq.do?sessionid=1362359014168174&source=target&seqID=TC198305)  [TC223310](http://plantgrn.noble.org/psRNATarget/getseq.do?sessionid=1362359014168174&source=target&seqID=TC223310)  [CV500596](http://plantgrn.noble.org/psRNATarget/getseq.do?sessionid=1362359014168174&source=target&seqID=CV500596)  [CK863467](http://plantgrn.noble.org/psRNATarget/getseq.do?sessionid=1362359014168174&source=target&seqID=CK863467) | Mevalonate disphosphate decarboxylase  Sulfate adenylyltransferase  Sulfate adenylyltransferase  Sulfate adenylyltransferase  Sulfate adenylyltransferase  Sulfate adenylyltransferase  Aspartic protease inhibitor 7 precursor  RE62247p  Histone H3 |
| miR4235 | [CK862691](http://plantgrn.noble.org/psRNATarget/getseq.do?sessionid=1362359826327893&source=target&seqID=CK862691) | Chromosome chr17 scaffold 16, whole genome shotgun sequence |
| miR437 | [TC197544](http://plantgrn.noble.org/psRNATarget/getseq.do?sessionid=1362360585208351&source=target&seqID=TC197544)  [TC205002](http://plantgrn.noble.org/psRNATarget/getseq.do?sessionid=1362360585208351&source=target&seqID=TC205002)  [TC199969](http://plantgrn.noble.org/psRNATarget/getseq.do?sessionid=1362360585208351&source=target&seqID=TC199969)  [CK251262](http://plantgrn.noble.org/psRNATarget/getseq.do?sessionid=1362360585208351&source=target&seqID=CK251262)  [TC195359](http://plantgrn.noble.org/psRNATarget/getseq.do?sessionid=1362360585208351&source=target&seqID=TC195359) | PEPC kinase 1b  BHLH transcription factor  Protein disulfide-isomerase precursor  Hypoxanthine phosphoribosyltransferase  Fertilization-independent endosperm protein |
| miR4372 | [TC210778](http://plantgrn.noble.org/psRNATarget/getseq.do?sessionid=1362360604149716&source=target&seqID=TC210778)  [CK274272](http://plantgrn.noble.org/psRNATarget/getseq.do?sessionid=1362360604149716&source=target&seqID=CK274272) | Chromosome chr15, whole genome shotgun sequence  Chromosome chr15, whole genome shotgun sequence |
| miR4394 | [CN515132](http://plantgrn.noble.org/psRNATarget/getseq.do?sessionid=1362360647727761&source=target&seqID=CN515132)  [TC195581](http://plantgrn.noble.org/psRNATarget/getseq.do?sessionid=1362360647727761&source=target&seqID=TC195581)  [CN513810](http://plantgrn.noble.org/psRNATarget/getseq.do?sessionid=1362360647727761&source=target&seqID=CN513810) | Aspartic protease inhibitor 8 precursor  Major intrinsic protein 2-like; n=1; *Solanum tuberosum*  Aspartic protease inhibitor 8 precursor |
| miR446 | [CX161930](http://plantgrn.noble.org/psRNATarget/getseq.do?sessionid=1362361135866765&source=target&seqID=CX161930)  [TC197992](http://plantgrn.noble.org/psRNATarget/getseq.do?sessionid=1362361135866765&source=target&seqID=TC197992)  [TC225558](http://plantgrn.noble.org/psRNATarget/getseq.do?sessionid=1362361135866765&source=target&seqID=TC225558)  [CV435232](http://plantgrn.noble.org/psRNATarget/getseq.do?sessionid=1362361135866765&source=target&seqID=CV435232)  [AM908900](http://plantgrn.noble.org/psRNATarget/getseq.do?sessionid=1362361135866765&source=target&seqID=AM908900) | Gibberellin 2-oxidase  3-dehydroquinate dehydratase / shikimate dehydrogenase isoform 2  Expressed protein  Tam3-transposase  Os12g0534000 protein |
| miR5170 | [TC204388](http://plantgrn.noble.org/psRNATarget/getseq.do?sessionid=1362361170544179&source=target&seqID=TC204388) | Mg protoporphyrin IX chelatase |
| miR837 | [BQ516625](http://plantgrn.noble.org/psRNATarget/getseq.do?sessionid=1362361580609732&source=target&seqID=BQ516625)  [CN513632](http://plantgrn.noble.org/psRNATarget/getseq.do?sessionid=1362361580609732&source=target&seqID=CN513632)  [TC223391](http://plantgrn.noble.org/psRNATarget/getseq.do?sessionid=1362361580609732&source=target&seqID=TC223391)  [TC201184](http://plantgrn.noble.org/psRNATarget/getseq.do?sessionid=1362361580609732&source=target&seqID=TC201184)  [TC197014](http://plantgrn.noble.org/psRNATarget/getseq.do?sessionid=1362361580609732&source=target&seqID=TC197014)  [TC210589](http://plantgrn.noble.org/psRNATarget/getseq.do?sessionid=1362361580609732&source=target&seqID=TC210589)  [CX700127](http://plantgrn.noble.org/psRNATarget/getseq.do?sessionid=1362361580609732&source=target&seqID=CX700127)  [TC221067](http://plantgrn.noble.org/psRNATarget/getseq.do?sessionid=1362361580609732&source=target&seqID=TC221067)  [TC196717](http://plantgrn.noble.org/psRNATarget/getseq.do?sessionid=1362361580609732&source=target&seqID=TC196717)  [TC226211](http://plantgrn.noble.org/psRNATarget/getseq.do?sessionid=1362361580609732&source=target&seqID=TC226211)  [BQ117105](http://plantgrn.noble.org/psRNATarget/getseq.do?sessionid=1362361580609732&source=target&seqID=BQ117105)  [TC215946](http://plantgrn.noble.org/psRNATarget/getseq.do?sessionid=1362361580609732&source=target&seqID=TC215946)  [TC226270](http://plantgrn.noble.org/psRNATarget/getseq.do?sessionid=1362361580609732&source=target&seqID=TC226270)  [TC214480](http://plantgrn.noble.org/psRNATarget/getseq.do?sessionid=1362361580609732&source=target&seqID=TC214480)  [CN514993](http://plantgrn.noble.org/psRNATarget/getseq.do?sessionid=1362361580609732&source=target&seqID=CN514993)  [TC201088](http://plantgrn.noble.org/psRNATarget/getseq.do?sessionid=1362361580609732&source=target&seqID=TC201088) | Host specificity protein J  Annexin p34-like protein-like  Annexin p34  Annexin p34  Annexin p34  Annexin p34  Annexin p34  TA4 protein  PFE18 protein  PFE18 protein  ANT-like protein  Expressed protein  T27G7.9  SGRP-1 protein  Proteinase inhibitor I  T27G7.9 |
| miR951 | [TC211976](http://plantgrn.noble.org/psRNATarget/getseq.do?sessionid=1362361670921174&source=target&seqID=TC211976)  [TC224932](http://plantgrn.noble.org/psRNATarget/getseq.do?sessionid=1362361670921174&source=target&seqID=TC224932) | Chromosome chr17, whole genome shotgun sequence  Chromosome chr14, whole genome shotgun sequence |
| **Expression pattern: up-regulation** | | |
| miRNAs | Target ID | Target annotations |
| miR1026 | [TC211408](http://plantgrn.noble.org/psRNATarget/getseq.do?sessionid=1362412606978549&source=target&seqID=TC211408)  [DV625431](http://plantgrn.noble.org/psRNATarget/getseq.do?sessionid=1362412606978549&source=target&seqID=DV625431)  [DN590265](http://plantgrn.noble.org/psRNATarget/getseq.do?sessionid=1362412606978549&source=target&seqID=DN590265) | S1 self-incompatibility locus-linked pollen 3.15 protein  Transposase for insertion sequence element IS640  TonB-dependent receptor precursor |
| miR1037 | [DR035698](http://plantgrn.noble.org/psRNATarget/getseq.do?sessionid=1362414254764862&source=target&seqID=DR035698)  [TC197209](http://plantgrn.noble.org/psRNATarget/getseq.do?sessionid=1362414254764862&source=target&seqID=TC197209)  [TC208061](http://plantgrn.noble.org/psRNATarget/getseq.do?sessionid=1362414254764862&source=target&seqID=TC208061)  [TC203631](http://plantgrn.noble.org/psRNATarget/getseq.do?sessionid=1362414254764862&source=target&seqID=TC203631) | UGT protein  Patatin-01 precursor  Extensin  Lysyl-tRNA synthetase |
| miR1039 | [TC210033](http://plantgrn.noble.org/psRNATarget/getseq.do?sessionid=1362414292705118&source=target&seqID=TC210033)  [TC223680](http://plantgrn.noble.org/psRNATarget/getseq.do?sessionid=1362414292705118&source=target&seqID=TC223680)  [TC195242](http://plantgrn.noble.org/psRNATarget/getseq.do?sessionid=1362414292705118&source=target&seqID=TC195242)  [EG013350](http://plantgrn.noble.org/psRNATarget/getseq.do?sessionid=1362414292705118&source=target&seqID=EG013350)  [BG096753](http://plantgrn.noble.org/psRNATarget/getseq.do?sessionid=1362414292705118&source=target&seqID=BG096753)  [TC195709](http://plantgrn.noble.org/psRNATarget/getseq.do?sessionid=1362414292705118&source=target&seqID=TC195709)  [TC197167](http://plantgrn.noble.org/psRNATarget/getseq.do?sessionid=1362414292705118&source=target&seqID=TC197167)  [TC196291](http://plantgrn.noble.org/psRNATarget/getseq.do?sessionid=1362414292705118&source=target&seqID=TC196291)  [TC208899](http://plantgrn.noble.org/psRNATarget/getseq.do?sessionid=1362414292705118&source=target&seqID=TC208899)  [TC203388](http://plantgrn.noble.org/psRNATarget/getseq.do?sessionid=1362414292705118&source=target&seqID=TC203388)  [TC205547](http://plantgrn.noble.org/psRNATarget/getseq.do?sessionid=1362414292705118&source=target&seqID=TC205547)  [AW906094](http://plantgrn.noble.org/psRNATarget/getseq.do?sessionid=1362414292705118&source=target&seqID=AW906094) | Beta-ketoacyl-ACP synthase I  Beta-ketoacyl-ACP synthase I  DS2 protein  Abscisic stress ripening protein  Abscisic stress ripening protein  DS2 protein  DS2 protein  DS2 protein  CDP-diacylglycerol-glycerol-3-phosphate3-phosphatidyltrans  BAC19.11  BAC19.11  U2 snRNP auxiliary factor |
| miR1048 | [TC198604](http://plantgrn.noble.org/psRNATarget/getseq.do?sessionid=1362414013257711&source=target&seqID=TC198604)  [TC221711](http://plantgrn.noble.org/psRNATarget/getseq.do?sessionid=1362414013257711&source=target&seqID=TC221711) | Ribose-phosphate pyrophosphokinase 1  Ferric-chelate reductase |
| miR1061 | [TC199564](http://plantgrn.noble.org/psRNATarget/getseq.do?sessionid=1362414894144298&source=target&seqID=TC199564)  [TC206978](http://plantgrn.noble.org/psRNATarget/getseq.do?sessionid=1362414894144298&source=target&seqID=TC206978) | Cell division cycle protein 48 homolog  Cell division control protein 48 homolog E |
| miR1222 | [BQ511009](http://plantgrn.noble.org/psRNATarget/getseq.do?sessionid=1362413650689194&source=target&seqID=BQ511009)  [TC216353](http://plantgrn.noble.org/psRNATarget/getseq.do?sessionid=1362413650689194&source=target&seqID=TC216353)  [TC219754](http://plantgrn.noble.org/psRNATarget/getseq.do?sessionid=1362413650689194&source=target&seqID=TC219754) | NB-ARC domain containing protein  F3F19.27 protein  Initiation factor eIF-4 gamma, middle |
| miR1511 | [CX162368](http://plantgrn.noble.org/psRNATarget/getseq.do?sessionid=1362413607038911&source=target&seqID=CX162368)  [BF053887](http://plantgrn.noble.org/psRNATarget/getseq.do?sessionid=1362413607038911&source=target&seqID=BF053887) | Gcap1 protein  Terminal protein |
| miR1522 | [TC215867](http://plantgrn.noble.org/psRNATarget/getseq.do?sessionid=1362413359928305&source=target&seqID=TC215867)  [TC206370](http://plantgrn.noble.org/psRNATarget/getseq.do?sessionid=1362413359928305&source=target&seqID=TC206370)  [TC200053](http://plantgrn.noble.org/psRNATarget/getseq.do?sessionid=1362413359928305&source=target&seqID=TC200053)  [TC197382](http://plantgrn.noble.org/psRNATarget/getseq.do?sessionid=1362413359928305&source=target&seqID=TC197382) | Probable cdp-diacylglycerol--glycerol-3-phosphate 3-phosphatidyltransferase transmembrane protein  Switch-associated protein 70 (SWAP-70)  Glutamine-fructose-6-phosphate transaminase 2  Extensin-like protein |
| miR1535 |  | No result |
| miR1919 | [DN588131](http://plantgrn.noble.org/psRNATarget/getseq.do?sessionid=1362412938069048&source=target&seqID=DN588131) | MSL1 protein |
| miR2083 | [TC206212](http://plantgrn.noble.org/psRNATarget/getseq.do?sessionid=1362412988072927&source=target&seqID=TC206212)  [CV476427](http://plantgrn.noble.org/psRNATarget/getseq.do?sessionid=1362412988072927&source=target&seqID=CV476427)  [TC210106](http://plantgrn.noble.org/psRNATarget/getseq.do?sessionid=1362412988072927&source=target&seqID=TC210106)  [TC199029](http://plantgrn.noble.org/psRNATarget/getseq.do?sessionid=1362412988072927&source=target&seqID=TC199029)  [DN849195](http://plantgrn.noble.org/psRNATarget/getseq.do?sessionid=1362412988072927&source=target&seqID=DN849195) | Zinc finger, RING-type  Ovule receptor-like kinase 28 precursor  At4g34360  At4g34360  WPP domain associated protein |
| miR2097 | [TC194647](http://plantgrn.noble.org/psRNATarget/getseq.do?sessionid=1362411353453282&source=target&seqID=TC194647)  [TC200366](http://plantgrn.noble.org/psRNATarget/getseq.do?sessionid=1362411353453282&source=target&seqID=TC200366)  [TC219627](http://plantgrn.noble.org/psRNATarget/getseq.do?sessionid=1362411353453282&source=target&seqID=TC219627) | Catechol oxidase B, chloroplast precursor  Elongation factor 1-alpha-like  BioY family protein |
| miR2101 | [CN463068](http://plantgrn.noble.org/psRNATarget/getseq.do?sessionid=1362411407657722&source=target&seqID=CN463068)  [TC197739](http://plantgrn.noble.org/psRNATarget/getseq.do?sessionid=1362411407657722&source=target&seqID=TC197739)  [TC224351](http://plantgrn.noble.org/psRNATarget/getseq.do?sessionid=1362411407657722&source=target&seqID=TC224351)  [TC217381](http://plantgrn.noble.org/psRNATarget/getseq.do?sessionid=1362411407657722&source=target&seqID=TC217381)  [TC223233](http://plantgrn.noble.org/psRNATarget/getseq.do?sessionid=1362411407657722&source=target&seqID=TC223233) | Aldose 1-epimerase  Sucrose-phosphate synthase  Homologue to UniRef100_Q38M69 Cluster: Ubiquitin carrier protein  Homologue to UniRef100_Q1HBA9 Cluster: Sucrose phosphate synthase  Homologue to UniRef100_P12360 Cluster: Chlorophyll a-b binding protein 6A, chloroplast precursor; n=1; *Solanum lycopersicum* |
| miR2119 | [DV624856](http://plantgrn.noble.org/psRNATarget/getseq.do?sessionid=1362411816210748&source=target&seqID=DV624856)  [DV626463](http://plantgrn.noble.org/psRNATarget/getseq.do?sessionid=1362411816210748&source=target&seqID=DV626463)  [CN515078](http://plantgrn.noble.org/psRNATarget/getseq.do?sessionid=1362411816210748&source=target&seqID=CN515078)  [TC222504](http://plantgrn.noble.org/psRNATarget/getseq.do?sessionid=1362411816210748&source=target&seqID=TC222504)  [CV492976](http://plantgrn.noble.org/psRNATarget/getseq.do?sessionid=1362411816210748&source=target&seqID=CV492976) | Proteinase inhibitor I  Cysteine protease inhibitor 9 precursor  Proteinase inhibitor 1 PPI2C4  Wound-induced proteinase inhibitor 1 precursor  YSL transporter 1 |
| miR2870 | [TC196250](http://plantgrn.noble.org/psRNATarget/getseq.do?sessionid=1362410852820519&source=target&seqID=TC196250)  [BE344149](http://plantgrn.noble.org/psRNATarget/getseq.do?sessionid=1362410852820519&source=target&seqID=BE344149)  [TC216159](http://plantgrn.noble.org/psRNATarget/getseq.do?sessionid=1362410852820519&source=target&seqID=TC216159)  [TC209615](http://plantgrn.noble.org/psRNATarget/getseq.do?sessionid=1362410852820519&source=target&seqID=TC209615) | DNA-binding protein  DNA-binding protein  PBng143  Eukaryotic translation initiation factor 4E |
| miR2922 | [CK251645](http://plantgrn.noble.org/psRNATarget/getseq.do?sessionid=1362410122292236&source=target&seqID=CK251645)  [TC196106](http://plantgrn.noble.org/psRNATarget/getseq.do?sessionid=1362410122292236&source=target&seqID=TC196106)  [CN515042](http://plantgrn.noble.org/psRNATarget/getseq.do?sessionid=1362410122292236&source=target&seqID=CN515042)  [TC194647](http://plantgrn.noble.org/psRNATarget/getseq.do?sessionid=1362410122292236&source=target&seqID=TC194647)  [TC210274](http://plantgrn.noble.org/psRNATarget/getseq.do?sessionid=1362410122292236&source=target&seqID=TC210274) | Histone-lysine N-methyltransferase ATXR5  Histone-lysine N-methyltransferase ATXR5  Aspartic protease inhibitor 1 precursor  Catechol oxidase B, chloroplast precursor  Danio rerio Rep: Zgc:56382 - Danio rerio |
| miR3437 | [AM908864](http://plantgrn.noble.org/psRNATarget/getseq.do?sessionid=1362410171450862&source=target&seqID=AM908864)  [TC194726](http://plantgrn.noble.org/psRNATarget/getseq.do?sessionid=1362410171450862&source=target&seqID=TC194726)  [CK860336](http://plantgrn.noble.org/psRNATarget/getseq.do?sessionid=1362410171450862&source=target&seqID=CK860336)  [AM906606](http://plantgrn.noble.org/psRNATarget/getseq.do?sessionid=1362410171450862&source=target&seqID=AM906606) | AtPH1-like protein  Chloroplast lipocalin  Similar to UniRef100_Q2RAK5 Cluster: expressed protein  Ribsomal protein S2e |
| miR3464 | [CV471519](http://plantgrn.noble.org/psRNATarget/getseq.do?sessionid=1362409738193585&source=target&seqID=CV471519)  [TC195303](http://plantgrn.noble.org/psRNATarget/getseq.do?sessionid=1362409738193585&source=target&seqID=TC195303) | Cyclic nucleotide-binding domain (CNMP-BD) protein  Transthyretin |
| miR3512 | [CV473378](http://plantgrn.noble.org/psRNATarget/getseq.do?sessionid=1362409839314503&source=target&seqID=CV473378)  [CV506881](http://plantgrn.noble.org/psRNATarget/getseq.do?sessionid=1362409839314503&source=target&seqID=CV506881)  [TC218237](http://plantgrn.noble.org/psRNATarget/getseq.do?sessionid=1362409839314503&source=target&seqID=TC218237) | Class S F-box protein  D-erythro-7,8-dihydroneopterin triphosphate epimerase  AvrPto-dependent Pto-interacting protein 3 |
| miR3513 | [CK246391](http://plantgrn.noble.org/psRNATarget/getseq.do?sessionid=1362365885206266&source=target&seqID=CK246391)  [TC205347](http://plantgrn.noble.org/psRNATarget/getseq.do?sessionid=1362365885206266&source=target&seqID=TC205347)  [TC207686](http://plantgrn.noble.org/psRNATarget/getseq.do?sessionid=1362365885206266&source=target&seqID=TC207686)  [TC226401](http://plantgrn.noble.org/psRNATarget/getseq.do?sessionid=1362365885206266&source=target&seqID=TC226401)  [BM113620](http://plantgrn.noble.org/psRNATarget/getseq.do?sessionid=1362365885206266&source=target&seqID=BM113620)  [TC198697](http://plantgrn.noble.org/psRNATarget/getseq.do?sessionid=1362365885206266&source=target&seqID=TC198697)  [TC198894](http://plantgrn.noble.org/psRNATarget/getseq.do?sessionid=1362365885206266&source=target&seqID=TC198894)  [TC196114](http://plantgrn.noble.org/psRNATarget/getseq.do?sessionid=1362365885206266&source=target&seqID=TC196114)  [TC207427](http://plantgrn.noble.org/psRNATarget/getseq.do?sessionid=1362365885206266&source=target&seqID=TC207427)  [TC199110](http://plantgrn.noble.org/psRNATarget/getseq.do?sessionid=1362365885206266&source=target&seqID=TC199110) | TGB2 protein  Dihydrodipicolinate synthase, chloroplast precursor  Tospovirus resistance protein C  36.4 kDa proline-rich protein  36.4 kDa proline-rich protein  36.4 kDa proline-rich protein  36.4 kDa proline-rich protein  Isoform 2 of Q9FHX5  Os04g0467400 protein  Chloroplast ATP synthase a chain precursor |
| miR4345 | [TC212946](http://plantgrn.noble.org/psRNATarget/getseq.do?sessionid=1362365677712484&source=target&seqID=TC212946) | Dolichol-phosphate mannosyltransferase-like (80) |
| miR4393 | [TC205927](http://plantgrn.noble.org/psRNATarget/getseq.do?sessionid=1362365368856507&source=target&seqID=TC205927)  [TC195350](http://plantgrn.noble.org/psRNATarget/getseq.do?sessionid=1362365368856507&source=target&seqID=TC195350)  [TC220356](http://plantgrn.noble.org/psRNATarget/getseq.do?sessionid=1362365368856507&source=target&seqID=TC220356) | GTP-binding Rop/Rac GTPase  Collagen alpha 1(XII) chain-like, skin and tendon  Os12g0516700 protein |
| miR472 | [NP919978](http://plantgrn.noble.org/psRNATarget/getseq.do?sessionid=1362364453387374&source=target&seqID=NP919978) [TC207242](http://plantgrn.noble.org/psRNATarget/getseq.do?sessionid=1362364453387374&source=target&seqID=TC207242)  [DN589262](http://plantgrn.noble.org/psRNATarget/getseq.do?sessionid=1362364453387374&source=target&seqID=DN589262)  [TC209328](http://plantgrn.noble.org/psRNATarget/getseq.do?sessionid=1362364453387374&source=target&seqID=TC209328)  [CN214760](http://plantgrn.noble.org/psRNATarget/getseq.do?sessionid=1362364453387374&source=target&seqID=CN214760)  [CX161378](http://plantgrn.noble.org/psRNATarget/getseq.do?sessionid=1362364453387374&source=target&seqID=CX161378)  [TC205058](http://plantgrn.noble.org/psRNATarget/getseq.do?sessionid=1362364453387374&source=target&seqID=TC205058) | Blight resistance protein SH10  WRKY DNA-binding protein  Preprotein translocase subunit secY, chloroplast precursor  Truncated RB  Blight resistance protein SH10  Preprotein translocase subunit secY, chloroplast precursor  Wiscott-Aldrich syndrome protein |
| miR5050 | [TC199256](http://plantgrn.noble.org/psRNATarget/getseq.do?sessionid=1362364933838358&source=target&seqID=TC199256)  [TC221710](http://plantgrn.noble.org/psRNATarget/getseq.do?sessionid=1362364933838358&source=target&seqID=TC221710)  [CK853109](http://plantgrn.noble.org/psRNATarget/getseq.do?sessionid=1362364933838358&source=target&seqID=CK853109)  [TC225374](http://plantgrn.noble.org/psRNATarget/getseq.do?sessionid=1362364933838358&source=target&seqID=TC225374) | Transcription initiation factor IIB-2  Glucose-1-phosphate adenylyltransferase  Vacuolar H^+^-pyrophosphatase  Glucose-1-phosphate adenylyltransferase small subunit, chloroplast precursor |
| miR5085 | [CK850974](http://plantgrn.noble.org/psRNATarget/getseq.do?sessionid=1362362879383045&source=target&seqID=CK850974)  [DN922860](http://plantgrn.noble.org/psRNATarget/getseq.do?sessionid=1362362879383045&source=target&seqID=DN922860)  [DN848784](http://plantgrn.noble.org/psRNATarget/getseq.do?sessionid=1362362879383045&source=target&seqID=DN848784) | Phytochelatin synthase  Methyl jasmonate esterase  Phytochelatin synthase |
| miR5301 | [CN215356](http://plantgrn.noble.org/psRNATarget/getseq.do?sessionid=1362362932226661&source=target&seqID=CN215356)  [TC197508](http://plantgrn.noble.org/psRNATarget/getseq.do?sessionid=1362362932226661&source=target&seqID=TC197508) | SY48B6A.6b  Y48B6A.6b |
| miR536 | [BQ511891](http://plantgrn.noble.org/psRNATarget/getseq.do?sessionid=1362363218677566&source=target&seqID=BQ511891)  [TC200336](http://plantgrn.noble.org/psRNATarget/getseq.do?sessionid=1362363218677566&source=target&seqID=TC200336)  [TC224040](http://plantgrn.noble.org/psRNATarget/getseq.do?sessionid=1362363218677566&source=target&seqID=TC224040) | CCAAT-box transcription factor-related  Aldehyde dehydrogenase  Aldehyde dehydrogenase |
| mir830 | [TC218328](http://plantgrn.noble.org/psRNATarget/getseq.do?sessionid=1362363238368974&source=target&seqID=TC218328)  [BF460457](http://plantgrn.noble.org/psRNATarget/getseq.do?sessionid=1362363238368974&source=target&seqID=BF460457)  [BQ045748](http://plantgrn.noble.org/psRNATarget/getseq.do?sessionid=1362363238368974&source=target&seqID=BQ045748)  [TC209020](http://plantgrn.noble.org/psRNATarget/getseq.do?sessionid=1362363238368974&source=target&seqID=TC209020) | Chlorophyll a-b binding protein 7, chloroplast precursor  RING-finger protein  RING-finger protein  RING-finger protein |
| miR865 | [TC221845](http://plantgrn.noble.org/psRNATarget/getseq.do?sessionid=1362363680855528&source=target&seqID=TC221845)  [CV504510](http://plantgrn.noble.org/psRNATarget/getseq.do?sessionid=1362363680855528&source=target&seqID=CV504510)  [CV470202](http://plantgrn.noble.org/psRNATarget/getseq.do?sessionid=1362363680855528&source=target&seqID=CV470202)  [BG096380](http://plantgrn.noble.org/psRNATarget/getseq.do?sessionid=1362363680855528&source=target&seqID=BG096380)  [TC218555](http://plantgrn.noble.org/psRNATarget/getseq.do?sessionid=1362363680855528&source=target&seqID=TC218555)  [TC201494](http://plantgrn.noble.org/psRNATarget/getseq.do?sessionid=1362363680855528&source=target&seqID=TC201494)  [TC215037](http://plantgrn.noble.org/psRNATarget/getseq.do?sessionid=1362363680855528&source=target&seqID=TC215037)  [EG0136](http://plantgrn.noble.org/psRNATarget/getseq.do?sessionid=1362363680855528&source=target&seqID=EG013667)67  [CV476590](http://plantgrn.noble.org/psRNATarget/getseq.do?sessionid=1362363680855528&source=target&seqID=CV476590)  [TC206096](http://plantgrn.noble.org/psRNATarget/getseq.do?sessionid=1362363680855528&source=target&seqID=TC206096)  [TC197625](http://plantgrn.noble.org/psRNATarget/getseq.do?sessionid=1362363680855528&source=target&seqID=TC197625)  [TC198447](http://plantgrn.noble.org/psRNATarget/getseq.do?sessionid=1362363680855528&source=target&seqID=TC198447)  [EG015505](http://plantgrn.noble.org/psRNATarget/getseq.do?sessionid=1362363680855528&source=target&seqID=EG015505) | Bromodomain-containing protein  SNADH dehydrogenase subunit 2  Bromodomain-containing protein  PK11-C5  PK11-C1  PK11-C1  PK11-C1  Malate dehydrogenase precursor  LOC447970 protein  GTP-binding nuclear protein Ran2  Predicted protein  SAG101  Zgc:100800 |
| miR952 | [TC199124](http://plantgrn.noble.org/psRNATarget/getseq.do?sessionid=1362364402033742&source=target&seqID=TC199124) | LOB domain-containing protein 37 |
