# Supplementary material for: Identification of Novel and Conserved MicroRNAs Related to Drought Stress in Potato by Deep Sequencing
Source: PLoS One. 2014 Apr 18;9(4):e95489. doi: 10.1371/journal.pone.0095489 (PMC3991665; doi:10.1371/journal.pone.0095489)
Supplement: Table S4 — Predicted target genes of sixty differentially expressed novel miRNAs and their functional annotation. (DOC) [file pone.0095489.s004.doc]

**Table S4 Predicted target genes of sixty differentially expressed novel miRNAs and their functional annotation**

| **Expression pattern: down-regulation** | | | |
| --- | --- | --- | --- |
| miRNAs | Target ID | | Target annotations |
| stu-miR-108 | TC221709  TC216907  TC217514  EG009962  TC214743  TC197704 | | YA1 - Petunia hybrida  YA6 - Petunia hybrida  YA5 - Petunia hybrida  YA6 - Petunia hybrida  CCAAT-binding transcription factor subunit B  YA3 - Petunia hybrida |
| stu-miR-139 | CK860770  TC209712  TC220318  TC224642 | | Os02g0678500 protein  Expressed protein  Zeta-carotene desaturase  NL0D |
| stu-miR-144 | CV431841  CV500557  CN215402  TC220614  TC212976 | | 0 day neonate kidney cDNA RIKEN full-length enriched library Clone:D630041G18 product  Chaperone protein dnaJ 11, chloroplast precursor  F19P19.16  Glutathione S-transferase  G2/mitotic-specific cyclin-1 |
| stu-miR-152 | CK851519  TC196603 | | ADP-ribosylation factor 2  50S ribosomal protein L12, chloroplast precursor |
| stu-miR-153 | CV503067  CK254088  TC202116  TC197501 | | Metal-dependent phosphohydrolase HD domain-containing protein  Metal-dependent phosphohydrolase HD domain-containing protein  Endo-1,4-beta-glucanase  Eukaryotic translation initiation factor 4E |
| stu-miR-155 | TC195849 | | Probable vacuolar proton pump subunit H |
| stu-miR-160 | TC226475  BQ505795  CV502155 | | Disease resistance protein RGA2  Mitochondrial carrier protein  Heat shock protein 70 |
| stu-miR-179 | TC196812  TC218101  TC214982 | | Neutral leucine aminopeptidase preprotein precursor  Chloroplast precursor  Neutral leucine aminopeptidase preprotein precursor |
| stu-miR-243 | TC206198  TC212634  CV302861  EG015055  BE343711  CX162317  TC199632 | | Monooxygenase  Integral membrane family protein  Peroxidase 1  Short chain dehydrogenase  Nodulin-like protein  Chloroplast 30S ribosomal protein S7  DNA ligase IV |
| stu-miR-25 | TC211510  DN587219  DV62315 | | Auxin-binding protein precursor  Os05g0366800 protein  Aspartic protease inhibitor 8 precursor |
| stu-miR-286 | CV498283  TC217913  BG887512  TC210326  TC201561  TC217268  AU279211 | | T1G11.9 protein  NADH-ubiquinone oxidoreductase chain 6  SJCHGC02683 protein  Calcineurin B-like protein  Eukaryotic translation initiation factor 3 subunit D  Virion infectivity factor  Alpha tubulin-2A |
| stu-miR-301 | TC206198  TC212634  CV302861  EG015055  BE343711  CX162317 | | Monooxygenase  Integral membrane family protein  Peroxidase 1  Short chain dehydrogenase  Nodulin-like protein  Chloroplast 30S ribosomal protein S7 |
| stu-miR-31 | TC206799  CV501547  TC206128  DN939167 | | Expressed protein  CC-NBS-LRR protein  Calmodulin-binding heat-shock protein  Cytochrome B561 precursor |
| stu-miR-342 | CV498283  TC217913  BG887512  TC210326  TC201561  TC217268  AU279211 | | T1G11.9 protein  NADH-ubiquinone oxidoreductase chain 6  SJCHGC02683 protein  Calcineurin B-like protein  Eukaryotic translation initiation factor 3 subunit D  Virion infectivity factor  Alpha tubulin-2A |
| stu-miR-364 | BQ513766  TC214908 | | 110 kDa 4SNc-Tudor domain protein-*Pisum sativum*  Serine protease-*Solanum lycopersicum* |
| stu-miR-383 | TC210527  TC218817  TC209641  TC205721 | | Chlamydomonas reinhardtii  Non-specific lipid-transfer protein 1 precursor  Uncharacterized protein F12A10.7 precursor  T23E18.21-*Arabidopsis thaliana* |
| stu-miR-4 | TC204728 | | Protein kinase |
| stu-miR-419 | TC219581  CN214050 | | 60S ribosomal protein L13a-like protein  Caffeoyl-CoA O-methyltransferase 5 |
| stu-miR-423 | TC196489  BE921788 | | Integral membrane family protein  Acyl carrier protein precursor |
| stu-miR-473 | CV302861  TC206198 | | Peroxidase 1  Monooxygenase |
| stu-miR-475 | TC203637  TC201282  TC196758 | | 26S proteasome subunit RPN7  Zinc finger protein 8  Vacuolar protein sorting-associated protein 28 homolog 1 |
| stu-miR-495 | TC199632  TC206198  TC209641  TC210527  TC195015 | | DNA ligase IV  Monooxygenase  Uncharacterized protein F12A10.7 precursor  Predicted protein  MATE efflux family protein precursor |
| stu-miR-513 | CX161318  TC214525  BQ519057  TC222017 | | Non-specific lipid-transfer protein 2 precursor  60S ribosomal protein L27  Elongation initiation factor 6  Probable UDP-glucose 6-dehydrogenase 1 |
| stu-miR-528 | GT888978  TC200801 | | 60S ribosomal protein L34  Cytochrome P450 71D7 |
| stu-miR-535 | TC222941  TC207727  TC222240 | | Os01g0562600 protein  Uncharacterized protein At5g28910.1  Myb domain-containing protein |
| stu-miR-574 | TC219236  CV302334  DN938732  TC199538  CK246274 | | Glycine-rich protein 2  Glycine-rich protein 2  Glycine-rich protein 2  Glycine-rich protein 2  Glycine-rich protein 2 |
| stu-miR-58 | BE919952  TC212184  TC213503  TC195933 | | Glucose acyltransferase  Glucose acyltransferase  At4g26550  DELLA protein GAI |
| stu-miR-585 | AM907171  TC212879  TC197221 | | Lycopene epsilon cyclase, chloroplast precursor  Ketol-acid reductoisomerase, chloroplast precursor  Chromosome undetermined scaffold 235 |
| stu-miR-66 | DV625909  DN938759  TC194595 | | Os02g0770100 protein  Alpha-glucan water dikinase, chloroplast precursor  Alpha-glucan water dikinase, chloroplast precursor |
| stu-miR-86 | TC214004 | | Uncharacterized protein At4g23470.3 |
| **Expression pattern: up-regulation** | | | |
| miRNAs | Target ID | Target annotations | |
| stu-miR-592 | TC204728  TC206826  TC222790  DN940457  TC215888 | Protein kinase  Apyrase 3  Apyrase GS50  F2P3.9 protein  Translation initiation factor IF-2 | |
| stu-miR-597 | CK853320  TC213393  TC224450  TC205571  CX699136  CV499132  DV624658 | GPI ethanolamine phosphate transferase  Uncharacterized protein At3g10770.2  Single-stranded nucleic acid binding R3H  Uncharacterized protein At3g18750.2  Single-stranded nucleic acid binding R3H  Phospholipid transfer protein-like protein  Cysteine protease inhibitor 8 precursor | |
| stu-miR-629 | TC197876 | Pyruvate kinase | |
| stu-miR-630 | TC220991 | Cytokinin-O-glucosyltransferase 3 | |
| stu-miR-643 | BQ519234  DV625529  BE341114  CN214516 | Barley protein Z homolog  Serpin  BEL1-related homeotic protein 11  P58IPK | |
| stu-miR-662 |  | No Result | |
| stu-miR-664 | BI919460  DN923319 | Type I restriction modification DNA specificity domain protein  Unknown protein | |
| stu-miR-668 | TC218678  TC205833  TC212324 | Mediator of RNA polymerase II transcription subunit 15  GS1-like protein  NB-ARC domain containing protein | |
| stu-miR-672 | BQ112675  TC221190  TC216442  TC207289  CK717261 | Predicted protein  MKS1  MKS1  GTP-binding protein TypA  Unknown | |
| stu-miR-680 | TC208401  BG589290  CK852653  TC218502  TC222389  BG600937  TC220322  TC218151  TC206955 | Cytochrome P450 4F3v2  *Yarrowia lipolytica* chromosome C of strain CLIB122  Tlr0801 protein  Cysteine protease  DNA methyltransferase  Multicystatin  Zinc metalloproteinase-like  Multicystatin  Unknown | |
| stu-miR-684 | TC214765  EG013665 | Whole genome shotgun sequence  Unknown | |
| stu-miR-700 | CV430620  TC206192  EG014104  TC219848 | Extensin  Cytokinin-regulated kinase 1  1-aminocyclopropane-1-carboxylate oxidase  Dinitrogenase reductase | |
| stu-miR-721 |  | No Result | |
| stu-miR-724 | AM908087  CN216184  TC208750  TC198545  TC225434  CK248934  TC216184  BG096704  TC216936 | MKIAA0885 protein  F18O14.14  F-box protein 7  Olfactory receptor 150  Nuclear RNA binding protein-like  Unknown  F-box protein 7  Multidrug resistance protein mdtO  Unknown | |
| stu-miR-727 | TC206223 | Unknown | |
| stu-miR-733 | TC215947 | Whole genome shotgun sequence | |
| stu-miR-742 | CV498856  CV302935  TC201520  CK718551  TC222562  TC196022  DN590130 | Plexin-A1 precursor  Superoxide dismutase [Fe]  Fe-superoxide dismutase  At2g04360  Spermatogenesis associated 3  Plastidic ATP/ADP-transporter  Unknown | |
| stu-miR-783 | CO502849  TC223777  TC201595  TC205893  TC197761  CK863443  TC204267  TC203206 | Cysteine proteinase 3 precursor  F6I1.12 protein  Tyramine hydroxycinnamoyl transferase  Branched-chain-amino-acid aminotransferase-like protein 2  Peptidyl-prolyl cis-trans isomerase  COG0086: DNA-directed RNA polymerase  AT3g53540/F4P12_240  MATE efflux family protein | |
| stu-miR-792 | CO502453  TC199930  DR034001 | T/G mismatch-specific endonuclease  Phosphatidylglycerol specific phospholipase C  Phosphatidylglycerol specific phospholipase C | |
| stu-miR-810 | TC203737 | Whole genome shot gun sequence | |
| stu-miR-842 | FG551268  CN514828 | Polyprotein  Aspartic protease inhibitor 8 precursor | |
| stu-miR-848 | BM11080  CK863609 | DSBA-like thioredoxin domain containing protein  Unknown | |
| stu-miR-856 | TC225708  DV627471  TC206268 | Mitogen-activated protein kinase 19  Kunitz-type enzyme inhibitor S9C11  Unknown | |
| stu-miR-860 | TC217219  TC204728  CV473917  TC222013  CN216363  CN215671  TC224966  TC197065 | Aspartic protease inhibitor 7 precursor  Protein kinase  Nodule-enhanced protein phosphatase type 2C  Fumarase  T7I23.17 protein  Fumarase  Mitogen-activated protein kinase  Similarity to lectin-like protein kinase | |
| stu-miR-861 | TC209546  CX162751  BQ046653  EG014256 | Monothiol glutaredoxin-S13  MADS11  Predicted protein  Unknown | |
| stu-miR-864 | TC201150  TC223668 | NADPH thioredoxin reductase  Unknown | |
| stu-miR-866 | CV502196 | DNA-binding protein phosphatase 2C | |
| stu-miR-886 | TC195695 | Whole genome shotgun sequence | |
| stu-miR-899 | TC216994  CK863110  TC196815  CK860200  EG013694  TC217533 | P58IPK  Transcription factor Myb1  Late blight resistance protein-like  Prf interactor 30137  Unknown  Unknown | |
| stu-miR-918 | TC212066  BI919460  TC216347  EG012040 | Type 1a plasma membrane receptor-like  Type I restriction modification DNA specificity domain protein  Glycine rich protein  Muramidase | |
